# Global emission trends of anthropogenic full-volatility-range organic compounds 1970–2020

**DOI:** 10.1093/nsr/nwag281

**Published:** 2026-05-18

**Authors:** Ruochong Xu, Ruqian Miao, Jingxian Li, Hanchen Ma, Hanwen Hu, Huaxuan Wang, Xizhe Yan, Xiaodong Liu, Dan Tong, Guannan Geng, Qi Chen, Kebin He, Qiang Zhang

**Affiliations:** Department of Earth System Science, Ministry of Education Key Laboratory for Earth System Modeling, Institute for Global Change Studies, Tsinghua University, Beijing 100084, China; State Key Laboratory of Regional Environment and Sustainability, College of Environmental Sciences and Engineering, Peking University, Beijing 100871, China; State Key Laboratory of Regional Environment and Sustainability, School of Environment, Tsinghua University, Beijing 100084, China; Department of Earth System Science, Ministry of Education Key Laboratory for Earth System Modeling, Institute for Global Change Studies, Tsinghua University, Beijing 100084, China; State Key Laboratory of Regional Environment and Sustainability, School of Environment, Tsinghua University, Beijing 100084, China; Department of Earth System Science, Ministry of Education Key Laboratory for Earth System Modeling, Institute for Global Change Studies, Tsinghua University, Beijing 100084, China; Department of Earth System Science, Ministry of Education Key Laboratory for Earth System Modeling, Institute for Global Change Studies, Tsinghua University, Beijing 100084, China; State Key Laboratory of Regional Environment and Sustainability, School of Environment, Tsinghua University, Beijing 100084, China; Department of Earth System Science, Ministry of Education Key Laboratory for Earth System Modeling, Institute for Global Change Studies, Tsinghua University, Beijing 100084, China; State Key Laboratory of Regional Environment and Sustainability, School of Environment, Tsinghua University, Beijing 100084, China; State Key Laboratory of Regional Environment and Sustainability, College of Environmental Sciences and Engineering, Peking University, Beijing 100871, China; State Key Laboratory of Regional Environment and Sustainability, School of Environment, Tsinghua University, Beijing 100084, China; Institute for Carbon Neutrality, Tsinghua University, Beijing 100084, China; Department of Earth System Science, Ministry of Education Key Laboratory for Earth System Modeling, Institute for Global Change Studies, Tsinghua University, Beijing 100084, China

**Keywords:** full-volatility range, L/S/IVOC emissions, long-term trends and drivers, organic aerosols

## Abstract

Full-volatility-range organic compounds (FVOC), including low-volatility, semi-volatile, intermediate-volatility, and non-methane volatile organic compounds (L/S/IVOC and NMVOC), are key components influencing atmospheric chemistry and climate. Accurate estimates of their emissions are crucial for modeling air quality and understanding aerosol budgets. However, the long-term trends of global FVOC emissions remain unquantified. Here, we develop a volatility-bin-resolved global anthropogenic FVOC emission inventory for 1970–2020 under the Multi-resolution Emission Inventory model for Climate and air pollution research (MEIC) framework (MEIC-global-FVOC), integrating measured gas- and particle-phase emission factors. The MEIC-global-FVOC emission inventory fills the gaps in current inventories by capturing IVOC emissions and likely accounting for part of the evaporative fraction of low-volatility and semi-volatile organic compound (L/SVOC) emissions, leading to 28%–41% higher estimates for global organic compound emissions than current inventories. Global FVOC emissions increase from 106.1 Mt to 165.0 Mt during 1970–2020, with higher growth rates of L/SVOC (9.7% per decade) and IVOC (11.5% per decade) than that of NMVOC (8.4% per decade), mainly driven by increased activities in scattered sources such as residential biofuel combustion and volatile chemical products (VCPs) use. Global L/S/IVOC emissions increase from 35.0 Mt to 58.4 Mt during 1970–2020. Regionally, L/S/IVOC emissions increase rapidly in developing regions due to rising biofuel combustion, whereas emissions in developed regions decrease or remain stable under the offsetting effects of improved PM controls, coal phase-out, sustained rural biofuel use, and increased VCPs demand. Despite notable uncertainties, the MEIC-global-FVOC emission inventory provides more complete estimates of global organic compound emissions and a new dataset for advancing chemical transport modeling and understanding air pollution sources.

## Teaser

This work develops a global anthropogenic emission inventory of full-volatility-range organic compounds for 1970-2020, filling the gaps in current inventories by capturing intermediate-volatility and semi-volatile organic compound emissions.

## INTRODUCTION

Organic compounds are the key components of the Earth’s atmosphere, influencing atmospheric chemistry, air quality, climate system, and human health [1–4]. Based on their saturation vapor concentration (C*), atmospheric organic compounds are typically classified as extremely-low- and low-volatility organic compounds (LVOC; C* < 0.3 μg/m^3^), semi-volatile organic compounds (SVOC; 0.3 μg/m^3^ < C* < 300 μg/m^3^), intermediate-volatility organic compounds (IVOC; 300 μg/m^3^ < C* < 3×10^6^ μg/m^3^), and volatile organic compounds (VOC; C* > 3×10^6^ μg/m^3^), spanning a wide volatility range [5].

The diversity and complexity of atmospheric organic compounds are a longstanding challenge to elucidate their composition, evolution, and environmental implications [4]. Early studies mainly focus on primary organic aerosols (POA; mainly comprising particle-phase LVOC and SVOC) and gas-phase VOC. However, research in the recent two decades indicate the important roles of SVOC and IVOC (S/IVOC) in the formation of secondary organic aerosols (SOA) [6–9]. A subset of LVOC, SVOC, and IVOC (L/S/IVOC) have also been identified as hazardous air pollutants (HAPs), for example, polycyclic aromatic hydrocarbons (PAHs), and per- and polyfluoroalkyl substances (PFAS), posing serious public health risks [3,10]. Therefore, accurate emission estimates of anthropogenic organic compounds with full-volatility coverage (i.e. full-volatility-range organic compounds, FVOC) are crucial for improving mass closure, understanding their atmospheric fates and impacts, and informing pollution mitigation strategies [4,9,11].

Traditional global and regional emission inventories have only included non-methane volatile organic compound (NMVOC) and POA emissions [12–16]. Some studies have estimated L/S/IVOC emissions by applying empirical SVOC-to-POA or IVOC-to-NMVOC ratios to existing POA or NMVOC emissions [7,17–20]. Their estimates have large uncertainties due to the lack of detailed volatility information and may introduce substantial biases due to inconsistent experimental conditions when determining the empirical ratios from field or laboratory measurements. Recent studies have utilized source-specific, volatility-bin-resolved emission factors to develop FVOC emission inventories with so-called complete volatility distributions [9,11,21]. One of them provides global FVOC emissions in 2015 on the basis of the ECLIPSE V6b dataset [21]. However, the long-term trends and spatiotemporal variations of global FVOC emissions have not yet been analyzed, hindering a thorough understanding of the long-term trends and impacts of organic aerosols (OA). In addition, uniform unabated emission factors are generally applied across regions, despite laboratory experiments indicating substantial regional differences [22–24]. More efforts are needed to develop long-term global FVOC emission inventories with improved regional representations, and to assess their reliability through comparisons between observations and updated OA modeling by chemical transport models (CTMs) [9,25].

Here, we address the gap in representing the long-term trends of global FVOC emissions by developing a new global anthropogenic FVOC emission inventory under the bottom-up framework of the Multi-resolution Emission Inventory model for Climate and air pollution research (MEIC-global-FVOC), which is built upon a recently developed global technology-based NMVOC emission inventory (MEIC-global-NMVOC) [26,27]. We integrate a set of measured gas- and particle-phase unabated emission factors from advanced laboratory experiments to estimate global L/S/IVOC emissions from 1970 to 2020 over nine volatility bins, thereby constituting a FVOC emission inventory together with the previously estimated NMVOC emissions. The long-term emission trends and drivers are analyzed across volatility bins, source sectors, and regions, highlighting the added value of the MEIC-global-FVOC inventory in capturing the emission changes of components missing from traditional emission inventories. We further compare our estimates with previous studies, identify the causes of discrepancies, and discuss the potential implications for understanding OA formation and mitigation. The robustness and applicability of the MEIC-global-FVOC emission inventory are supported by good agreements between the modeled OA concentrations in GEOS-Chem and observations across regions, as briefly summarized in this study and presented in detail by our companion paper (unpublished data).

## RESULTS

### Addressing the gap in global FVOC emissions 1970–2020

**Figure 1.**
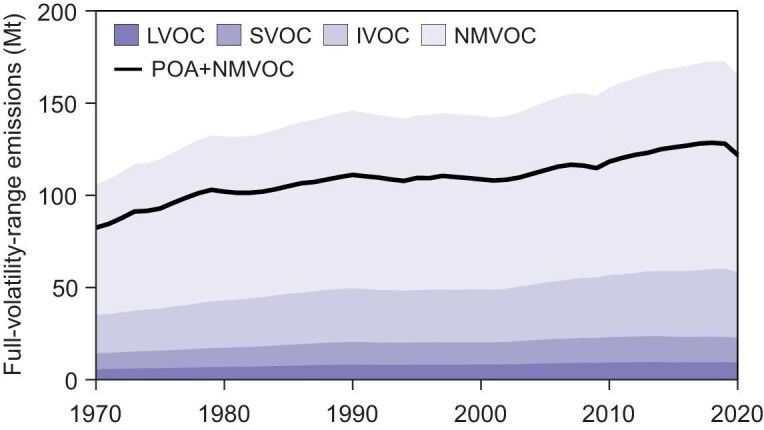
Global FVOC emission trends from 1970 to 2020, aggregated by volatility bin (LVOC, SVOC, IVOC, and NMVOC). The black solid line represents the sum of global POA and NMVOC emissions [13,27]. OC emissions from CEDS are converted to POA using an OM-to-OC ratio of 1.4.

**Figure 2.**
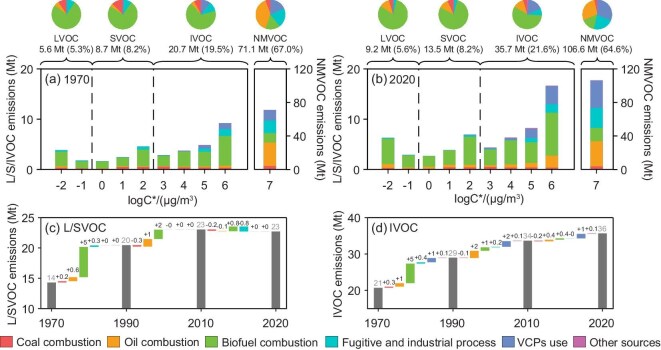
(a and b) Global FVOC emissions by volatility bin, accumulated by major sector in (a) 1970 and (b) 2020. The pie charts show the sectoral shares of LVOC, SVOC, IVOC, and NMVOC emissions. (c and d) Sectoral contributions to global (c) L/SVOC and (d) IVOC emission changes from 1970 to 2020. The colored bars in (c and d) represent the cumulative sectoral emission changes over the given periods. The sector mapping is presented in Table S1.

Global total emissions of anthropogenic FVOC increase from 106.1 Mt in 1970 to 172.6 Mt in 2019, before declining by 4.4% in 2020 due to the COVID-19 pandemic (Fig. 1). NMVOC emissions account for the majority of FVOC emissions (64%–68%), followed by IVOC (19%–22%), SVOC (8%–9%), and LVOC (5%–6%) (Figs 1 and 2a, b). During 1970–2020, the shares of NMVOC emissions slightly decrease within the full-volatility range, accompanied by a modest increase in IVOC emission shares, while the shares of LVOC and SVOC emissions remain stable.

The FVOC emissions here address the missing components in traditional emission inventories that only consider POA and NMVOC, thereby providing a more complete representation of organic compound emissions. During 1970–2020, global FVOC emissions are 28%–41% higher than the sum of POA and NMVOC emissions (Fig. 1 and Fig. S1) [12,13,27]. Specifically, IVOC emissions, which are missing from traditional emission inventories, account for ∼20% of global FVOC emissions. Compared with POA emissions, global LVOC and SVOC (L/SVOC) emissions are also 26%–109% higher. One possible reason is that part of the evaporative fraction of L/SVOC emissions under ambient conditions may be represented [6,11,17]. Alternatively, the differences may arise from variations in the measurements of POA and L/SVOC emission factors or other methodological differences. Importantly, the emission growth rates of L/SVOC and IVOC (9.7% and 11.5% per decade during 1970–2020) exceed those of POA (6.1% per decade) and NMVOC (8.4% per decade). This leads to a widening gap between full-volatility-range and traditional emission inventories over time, revealing the emerging importance of the newly developed inventory.

From a volatility-bin-resolved perspective, global anthropogenic FVOC emissions exhibit asymmetric W-shaped volatility distributions (Fig. 2a, b and Fig. S2). Within the L/S/IVOC range, the distributions are skewed towards IVOC emissions, with peaks at log_10_C* bins of ≤−2, 2, and 6. Oil combustion, fugitive and industrial process, and the use of volatile chemical products (VCPs) are the major contributors to NMVOC emissions (log_10_C* bin of ≥7). In contrast, biofuel combustion dominates L/S/IVOC emissions across all volatility bins (49%–83%). Fugitive and industrial process and the use of VCPs become increasingly important in higher-volatility IVOC bins, jointly contributing 26%–33% of emissions in the log_10_C* bins of 5 and 6.

### Sectoral contributions to global emission trends

As NMVOC emissions have been analyzed across multiple dimensions in our previous work [27], the following analysis primarily focuses on the sectoral contributions to L/S/IVOC emission trends. Global L/SVOC and IVOC emissions increase substantially by 43% and 40% during the period of 1970–90, respectively (Fig. 2c, d). Thereafter, L/SVOC emissions further increase to 23.0 Mt by 2010 and then remain stable, while IVOC emissions grow steadily to 36.9 Mt by 2019 before declining by 3% in 2020.

Biofuel combustion dominates global L/S/IVOC emission growth before 1990, contributing more than 80% and 60% of L/SVOC and IVOC emission increases, and remains a major driver thereafter (Fig. 2c, d). During 1990–2010, the contribution of oil combustion becomes increasingly comparable to that of biofuel combustion. After 2010, the plateau in global L/SVOC emissions reflects the offset between emission decreases from fugitive and industrial process—mainly due to strengthened PM controls—and continued increases from biofuel combustion. In contrast, global IVOC emission growth since 2010 has been primarily driven by emission increases from VCPs use.

**Figure 3.**
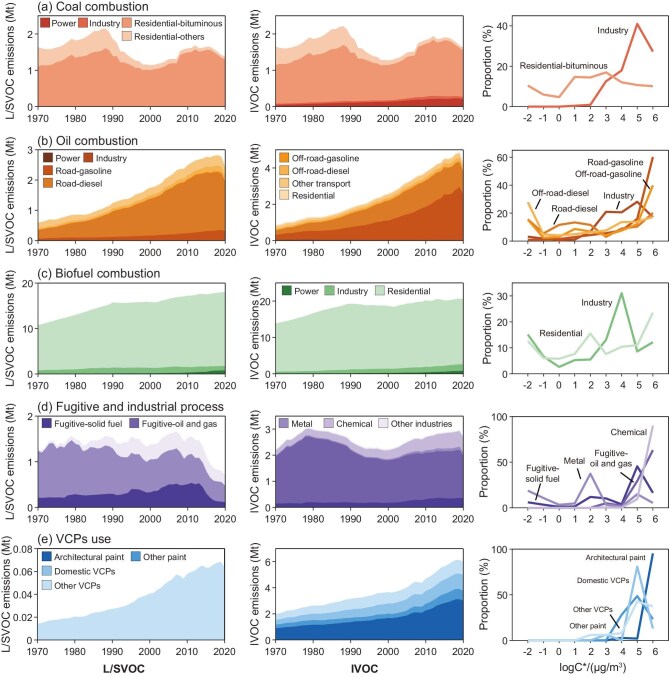
Global L/SVOC (left column) and IVOC (middle column) emissions from 1970 to 2020, accumulated by detailed source for (a) coal combustion, (b) oil combustion, (c) biofuel combustion, (d) fugitive and industrial process, and (e) VCPs use. The right column shows normalized volatility distributions of L/S/IVOC emissions in 2020 for representative sources. The sector mapping is presented in Table S1.

Global L/SVOC and IVOC emissions from different sources are further illustrated for each of the major sectors (Fig. 3). Residential coal combustion dominates coal-related emissions, showing a nadir between 1990 and 2010 as the rising consumption in developing countries is counterbalanced by concurrent declines in developed countries (Fig. 3a). Its volatility distribution is more uniform than that of industrial coal combustion, reflecting differences in combustion conditions and the effects of end-of-pipe PM controls in industries that remove most of the L/SVOC emissions.

For oil combustion, on-road diesel vehicles are the predominant driver of L/SVOC emission growth, the contributions of which increase from 47% in 1970 to 67% in 2020 (Fig. 3b). In contrast, IVOC emissions from on-road diesel and gasoline vehicles are comparable during 1970–2010, but the emissions from on-road gasoline vehicles increase steeply after 2010, mainly due to the rapid expansion of emission-intensive motorcycle fleets in emerging economies [28]. Regarding volatility distributions, diesel and gasoline vehicles show higher proportions in the L/SVOC and IVOC ranges, respectively, reflecting differences in fuel compositions and engine types [29]. Similar to coal combustion, the residential sector dominates emissions from biofuel combustion and exhibits a distinct volatility distribution compared with that of the industry sector (Fig. 3c).

For fugitive and industrial process, emissions from solid fuel production and metal industry account for the majority (68%–97%) of L/SVOC emissions, which drive the decreasing trend during 2010–20 due to strengthened emission controls (Fig. 3d). In contrast, fugitive emissions from oil and gas production are the predominant driver of IVOC emission changes. The volatility distributions of chemical industry and oil and gas production are highly concentrated in the IVOC range, whereas those of metal industry and solid fuel production exhibit asymmetric W-shaped patterns. L/SVOC emissions from the use of VCPs are minor and mainly originate from the use of glues, adhesives, and pesticides (Fig. 3e). The use of architectural paints represent the largest contributor (53%) to IVOC emission growth during 1970–2020, followed by domestic VCPs use. The volatility distributions of VCPs-related sources are strongly skewed towards the log_10_C* bins of 5 and 6.

### Regional emission trends

**Figure 4.**
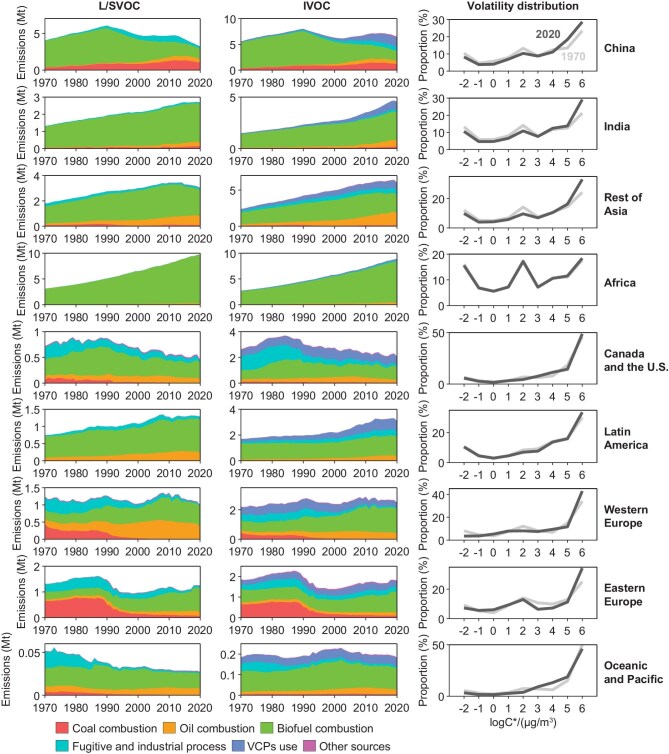
Regional L/SVOC (left column) and IVOC (middle column) emissions from 1970 to 2020 accumulated by major sector. The right column shows normalized volatility distributions of regional L/S/IVOC emissions in 1970 (light grey lines) and 2020 (dark grey lines). The region mapping is presented in Table S2.

Figure 4 shows the regional L/SVOC and IVOC emissions during 1970–2020. The regional grouping considers both the level of economic development and geographical proximity (see Text S3 and Table S2 for details). In developed regions such as Canada and the U.S. and Western Europe, L/SVOC and IVOC emissions either decrease or remain stable. These trends are driven by reduced contributions from coal combustion and industrial process, steady or rising contributions from biofuel and oil combustion, and substantial VCPs-related IVOC emissions. The patterns are shaped by the combined effects of residential coal phase-out, improved industrial PM controls, persistent rural biofuel consumption, and growing demand for VCPs [27,30,31].

In developing regions, emissions follow different trajectories. In India and Africa, L/SVOC and IVOC emissions double or even triple during 1970–2020, primarily driven by increased residential biofuel combustion for cooking and heating. In China, L/SVOC and IVOC emissions peak around 1990 and decline during 1990–2000 due to reduced biofuel combustion [32]. During 2000–10, L/SVOC emissions remain stable while IVOC emissions slightly increase, reflecting the offsetting trends between decreased emissions from biofuel combustion and increased emissions from other sectors (e.g. coal combustion and VCPs use). Both emissions in China decline during 2010–20, driven by stringent clean air actions [33]. In Eastern Europe, L/SVOC and IVOC emissions decline substantially after the dissolution of the Former Soviet Union in the 1990s, and show slight increases during 2000–20. Over this period, the predominant emission contributor shifts from coal combustion to biofuel combustion.

Volatility distributions vary across regions. In developing regions, volatility distributions generally exhibit W-shaped patterns, whereas in developed regions, they are more skewed towards the IVOC range, which is consistent with higher contributions from VCPs use and wood combustion [24,31]. From 1970 to 2020, volatility distributions remain relatively stable in developed regions and several developing regions (e.g. Africa), indicating consistent emission structures. However, regions such as China and India show increased shares in the IVOC bins, driven by sustained growth in VCPs-related emissions. This may reflect increased proportions of gas-phase emissions (Fig. S3), and consequently, more precursors for secondary pollution formation [34].

Figure S4 further illustrates the regional contributions to main L/SVOC and IVOC sources within the major sectors. Eastern Europe is the predominant contributor to both L/SVOC and IVOC emissions from residential coal combustion in 1970. After the dissolution of the Former Soviet Union, contributions from Eastern Europe decline sharply, while those from China and India increase substantially. For residential biofuel combustion, Africa gradually replaces China as the largest contributor to L/SVOC and IVOC emissions, driven by population growth and heavy reliance on primary biofuels.

Canada and the U.S., Western Europe, and Eastern Europe together account for 52% and 45% of L/SVOC and IVOC emissions from on-road gasoline vehicles in 1970, respectively. Their contributions decline substantially thereafter, while rest of Asia emerges as the largest contributor by 2020, accounting for ∼50% of L/SVOC and IVOC emissions, boosted by the expansion of low-emission-standard vehicle fleets in South and Southeast Asia [28]. A similar shift in predominant contributors from developed to developing regions is observed for emissions from off-road diesel vehicles, although developed regions still account for notable shares in 2020. In contrast, IVOC emissions from oil and gas production exhibit relatively balanced regional contributions throughout most of the 1970–2020 period.

Different VCPs application categories show distinct trends of regional contributions. For the use of architectural paints, developed regions contribute over half of IVOC emissions in 1970. However, the regional distribution becomes more balanced over time, with five regions each accounting for 14%–23% of emissions by 2020. In contrast, for the use of other paints, China’s contribution increases markedly after 2000 and reaches 67% of IVOC emissions by 2020, fueled by the rapid expansion of paint-intensive industries.

### Drivers of emission growth

As shown above, L/SVOC and IVOC emissions exhibit more pronounced growth than NMVOC during 1970–2020, leading to a widening gap between full-volatility-range and traditional emission inventories. To further clarify the underlying causes, we perform a driving factor decomposition analysis following the approach in our previous work [27]. The effects of two driving factors, changes of activity rates and other emission parameters (i.e. the combination of technology distributions, unabated emission factors, and emission control) are separated here.

**Figure 5.**
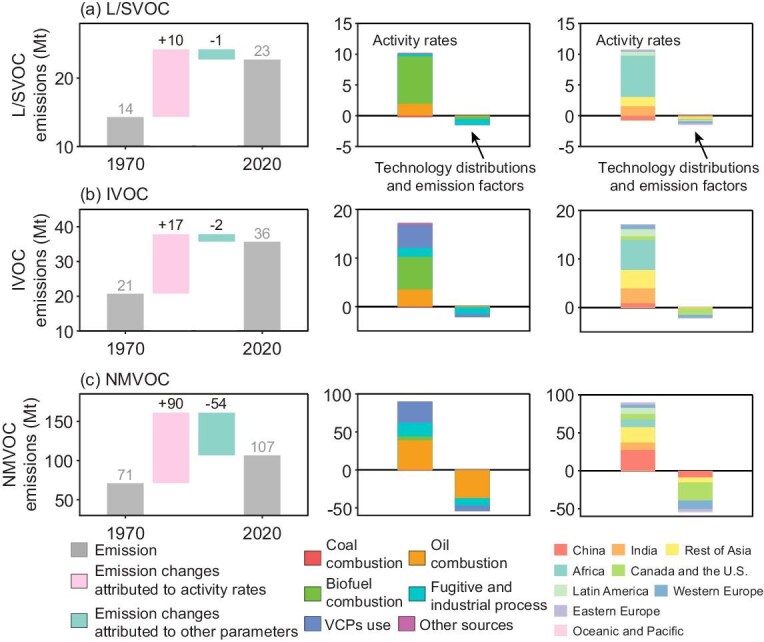
Driving factors of global emission changes attributed to activity rates (pink bars) and other parameters (i.e. the combination of technology distributions, unabated emission factors, and emission control; green bars) from 1970 to 2020 for (a) L/SVOC, (b) IVOC, and (c) NMVOC. Sectoral and regional contributions are shown in the middle and right panels, respectively.

Globally, the increases in L/SVOC and IVOC emissions are predominately driven by activity rate growth, while the effects of other parameters (e.g. technology improvement and emission control) are minimal (Fig. 5a, b). This contrasts with NMVOC emissions, for which changes in technology distributions and emission factors substantially slow the upward trend (Fig. 5c) [27,35,36]. The dominant role of activity rate growth for L/SVOC and IVOC emissions mainly arises from biofuel combustion and VCPs use, which are highly scattered in residential households and/or difficult to mitigate due to technical and economic barriers [27]. Mitigation measures targeting NMVOC emissions—such as reduction of oil product evaporation, end-of-pipe control of flue gases, and substitution of solvent-based VCPs—are only partially effective and less applicable to L/S/IVOC sources [14,36–38], leading to the faster growth of L/S/IVOC emissions within the full-volatility range during 1970–2020.

Developing regions, particularly Africa, India, and rest of Asia, contribute substantially to the activity rate growth that drives L/SVOC and IVOC emission increases, mainly due to growing residential biofuel combustion. In contrast, for NMVOC emissions, China’s contribution to activity rate growth becomes more prominent (right panel in Fig. 5 and Fig. S5). In developed regions such as Canada and the U.S. and Western Europe, mitigation measures targeting fine particulate matter (PM_2.5_) and NMVOC emissions exert synergistic effects in reducing L/SVOC and IVOC emissions, nearly offsetting the increases driven by activity rate growth. The results here highlight that the design of mitigation strategies should consider FVOC rather than NMVOC alone, to enhance synergistic reductions across volatility bins and improve the cost-effectiveness of mitigating secondary air pollution.

## DISCUSSIONS

### Comparison with previous studies across full-volatility range

A detailed comparison of NMVOC emissions across different studies is provided in Xu *et al.* (2025) [27]. The results show that NMVOC emission estimates in MEIC-global-FVOC are generally comparable to other emission inventories at aggregated level for global and regional totals, but notable differences remain in a few sectors, such as VCPs use [27]. Here, we focus on comparisons of L/S/IVOC emissions in this study (referred to as MEIC-global) and the volatility-resolved shares within FVOC emissions with previous studies at both global [7,17,21,39] and regional scales [9,19,25,40–46].

**Figure 6.**
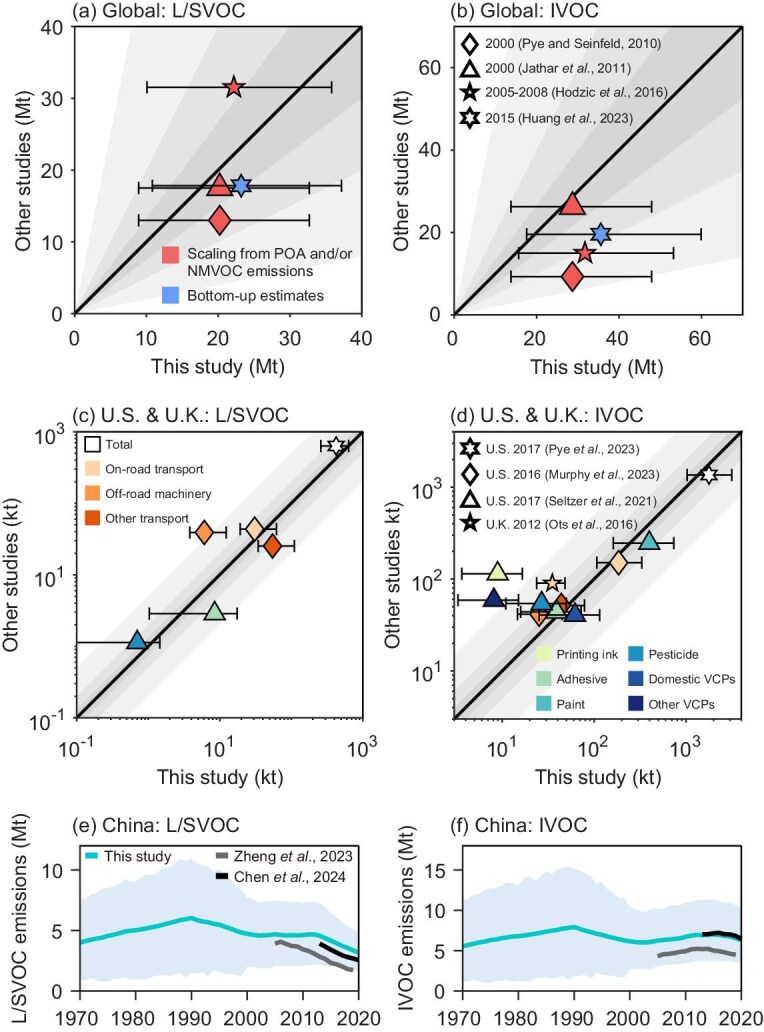
Comparison of L/SVOC (left column) and IVOC (right column) emissions between this study and other estimates (a and b) at global scale, (c and d) in the U.S. and the U.K., and (e and f) in China. Error bars in (a–d) and light blue shaded areas in (e and f) represent the uncertainty ranges of emission estimates. The solid black lines in (a–d) represent the 1:1 line, and the grey shaded areas represent 4:3 (3:4), 2:1 (1:2), and 5:1 (1:5) ranges, respectively. Note that the minor sources not included in this study (e.g. waste treatment) are excluded from other estimates to ensure comparison within the consistent boundary. Panel (e) and (f) present data only for Chinese mainland to ensure consistency among studies.

As shown in Fig. 6a, although falling within the uncertainty range (see Text S1 for a detailed uncertainty analysis), global L/SVOC emissions in MEIC-global for the year of 2015 are 30% higher than the emissions estimated by Huang *et al.* (2023) using a similar bottom-up approach [21]. The relative differences between our estimates and the empirical-ratio-based estimates of global L/SVOC emissions range from −30% to +55%. For global IVOC emissions, MEIC-global estimates are higher by 9%–209% than those estimated by previous studies (Fig. 6b). Different emission estimates may result from inconsistencies in activity data sources, emission factor treatments, and emission control representations as well as in the baseline emissions used in empirical-ratio-based approaches. For instance, Huang *et al.* (2023) use activity rates from the ECLIPSE V6b dataset and apply uniform unabated emission factors across regions [21], whereas we use activity rates from data fusion approaches and region-specific emission factors when available. When applying the higher emissions from MEIC-global to CTM simulations, modeled OA concentrations are still slightly or moderately lower than the observations (see Text S4, and our companion paper (unpublished data)), suggesting that the higher L/S/IVOC emission estimates in MEIC-global are likely more reasonable than the previous estimates.

Within the full-volatility range, Huang *et al.* (2023) report that LVOC, SVOC, IVOC, and NMVOC account for 6.9%, 4.8%, 15.3%, and 73.0% of global total emissions for the year of 2015. These global volatility-resolved contributions are broadly consistent with MEIC-global (Fig. 2a, b), although MEIC-global presents higher IVOC shares and lower NMVOC shares, indicating overall agreement in global volatility distributions.

For the U.S., the relative differences between MEIC-global and US EPA’s estimates are within ±35% for national total L/SVOC and IVOC emissions in 2017 (Fig. 6c, d) [25]. For most large sources (i.e. annual emissions >10 kt for L/SVOC and >50 kt for IVOC), such as on-road transport and paint use, the differences range from −29% to +62%. The discrepancies are more pronounced for minor sources. The MOVES (MOtor Vehicle Emission Simulator) and VCPy models used in EPA’s emission estimates likely incorporate more detailed technology- and species-specific data [40,41], which are difficult to represent in global-scale studies. For the UK, MEIC-global estimates for IVOC emissions from diesel vehicles are 61% lower than those estimated by Ots *et al.* (2016) [42], likely due to differences in methodologies.

For China, MEIC-global estimates for national total L/SVOC and IVOC emissions are generally consistent with those estimated by Chen *et al.* (2024) [9], but are 14%–94% and 33%–46% higher than the L/SVOC and IVOC emissions from Zheng *et al.* (2023) [43], respectively (Fig. 6e, f). These studies show consistent trends, although Zheng *et al.* (2023) indicate an earlier decline in L/SVOC emissions before 2010 [43]. Discrepancies are more pronounced at the sectoral level, particularly for transport and VCPs-related sources (Fig. S6), reflecting greater uncertainties in sectoral L/S/IVOC emission estimates.

Additionally, we compare the MEIC-global estimates with country-level emissions from Huang *et al.* (2023) [21], although emissions from open biomass burning could not be excluded in their available country-level data (Fig. S7). The country-level emission estimates are overall comparable between the two studies. However, substantial differences are observed in developing countries in Africa, Asia, and Latin America, likely due to the inclusion of open biomass burning in their estimates and/or methodological inconsistencies.

### Implications for OA modeling and understanding OA formation

Previous studies have shown that CTM simulations based on traditional POA emission inventories, which treat POA as non-volatile, may reproduce or overestimate observation-derived surface POA concentrations while often underestimate SOA concentrations [20,47]. In MEIC-global, the particle-phase fractions of L/S/IVOC emissions vary with environmental conditions that affect gas-particle partitioning, likely providing more gas-phase precursors for SOA formation. To illustrate the potential implications on OA modeling, we compare particle-phase L/S/IVOC emissions in MEIC-global under two representative conditions, ambient conditions and emission outlets, with the non-volatile POA emissions estimated by two widely used emission inventories, the Emission Database for Global Atmospheric Research (EDGAR) and the community emission data system (CEDS) [12,13]. Under ambient conditions, OA concentrations are region-specific average values derived from field observations (3.0–48.6 μg/m^3^) [21], whereas at emission outlets, OA concentrations are assumed to be 1000 μg/m^3^, representing the high-concentration environment immediately after organic compounds are emitted from sources. At emission outlets, L/S/IVOC are more likely condense onto particle surfaces, resulting in higher particle-phase fractions (i.e. POA emissions).

The results show that under ambient conditions, global particle-phase L/S/IVOC emissions from MEIC-global, accounting for about 27% of total L/S/IVOC emissions, are comparable to the non-volatile POA emissions from CEDS and EDGAR (Fig. S8). However, under high-OA-concentration conditions (i.e. as emitted), up to 45% of L/S/IVOC emissions partition to the particle phase, and MEIC-global emissions are 39%–63% and 48%–119% higher than those from CEDS and EDGAR, respectively. Regionally, the differences are larger in China, Africa, and Western Europe, but less evident in regions such as Canada and the U.S. Since the exact conditions under which the POA emission factors used by EDGAR and CEDS are measured are not available, the differences should be interpreted on a case-by-case basis. If most of the POA emission factors used by CEDS and EDGAR are measured under low- or moderate-concentration conditions, most of the evaporative fraction may be lost during dilution, whereas the full-volatility-range emission factors used by MEIC-global may capture at least part of the evaporative fraction of L/S/IVOC emissions, leading to higher emissions under emission-outlet conditions. By contrast, if most POA emission factors are measured under high-concentration conditions, the discrepancies may arise from differences in emission factor measurement techniques and other methodological differences. Further evaluation with volatility-resolved observational data is needed in future studies.

To address the gap in traditional emission inventories, empirical-ratio-based FVOC emission estimates are widely used as CTM inputs to quantify OA sources, budgets, and associated environmental effects [7,17,18,39,48], but may introduce substantial uncertainties and biases [11]. To illustrate the potential influence of different emission inputs on OA modeling, we further compare the MEIC-global emissions with estimates derived from applying different sets of empirical ratios used in previous studies [7,9,18,19]. These ratios are applied to NMVOC emissions from MEIC [27] and/or POA emissions from CEDS [13] to estimate L/S/IVOC emissions.

Comparisons show that empirical-ratio-based estimates may be substantially biased, although they mostly fall within the uncertainty ranges of MEIC-global emissions (Fig. S9). For instance, the small L/SVOC-to-POA ratios used by Zhao *et al.* (2016) [18] result in global L/SVOC emissions that are 49%–58% lower than those in MEIC-global, while the large IVOC-to-POA ratios lead to 24%–64% higher IVOC emissions before 2010. In contrast, the empirical ratios from Hodzic *et al.* (2016) [7] result in opposite biases. Even when empirical-ratio-based approaches produce consistent total estimates, they often misrepresent sectoral contributions. For example, IVOC emissions derived by scaling POA emissions neglect the contributions from industrial process and VCPs use [19], while scaling NMVOC emissions tends to underestimate the contributions from biofuel combustion [9]. Discrepancies are even larger at the regional scale (Fig. S10), indicating that empirical ratios should not be applied uniformly across regions. Such emission biases can propagate into OA modeling [11,18,49]. First, OA concentrations may be overestimated or underestimated in CTMs, limiting a quantitative understanding of OA pollution. Second, misrepresented sectoral contributions may misguide source apportionment and formulation of targeted mitigation strategies. These issues highlight the importance of volatility-bin-resolved FVOC emission estimates developed from measured gas- and particle-phase emission factors as a robust foundation for CTM simulations and mitigation strategy design.

We further apply the GEOS-Chem CTM to evaluate the robustness of MEIC-global-FVOC by comparing simulated results with observations. Details of the model configuration, diagnostics, and OA budget analysis are presented in our companion paper (unpublished data), with a brief analysis given here. Using MEIC-global-FVOC as emission input, simulated organic carbon (OC), POA, and SOA concentrations show generally good agreement with observations worldwide (see Text S4 for details). The new emission inventory enables GEOS-Chem to reproduce not only total OA but also the relationships between primary and secondary components, showing improved performance compared with many previous studies [7,17,39,48]. We further compare model performance with two additional simulations using CEDS, a widely-used traditional emission inventory [13], and CEDS with default IVOC emissions implemented in the GEOS-Chem ComplexSOA scheme [17]. The simulation driven by MEIC-global-FVOC exhibits improved overall performance, particularly by substantially reducing the SOA underestimation found in the other two simulations. These differences partly result from the representation of L/SVOC emissions in MEIC-global-FVOC, which can partition between gas and particle phases and thus provide additional SOA precursors, as well as inclusion of more accurate IVOC emission estimates. The improved modeling may provide new insights into OA sources and the formation of secondary air pollution. While mitigation of SOA pollution has typically focused on reducing NMVOC emissions [14,33], the full-volatility-range perspective indicates that L/S/IVOC emissions, the sectoral contributions and drivers of which differ from those of NMVOC (see Sections Sectoral contributions to global emission trends and Drivers of emission growth), play a non-negligible role in SOA formation and should be considered to improve the effectiveness of mitigation strategies [9,11,20].

This study has several limitations (see Text S1 for detailed discussions). First, the availability of measured gas- and particle-phase emission factors remains limited. In regions lacking local measurements, emission factors adopted from other regions may not be sufficiently representative of local conditions. This may introduce additional biases for regional emission analyses and OA modeling in CTMs. Second, the control efficiencies for L/SVOC and IVOC are assumed to be equivalent to those of PM_2.5_ and NMVOC, which may not fully capture the effects of control technologies or environmental conditions at emission outlets. Third, emissions from open biomass burning and waste treatment are not included, which may be important in certain regions. More complete source categories and more refined representations of emission parameters should be considered in future studies. Additionally, sensitivity analyses are conducted to evaluate the effects of emission control assumptions, volatility-bin definitions, and the exclusion of 2020—a year affected by the COVID-19 pandemic—on full-volatility-range emissions trends (see Text S3 for details). The results show that most of the quantitative and comparative findings remain robust in these sensitivity tests, supporting the reliability of the main conclusions.

In this study, we address the gap in representing long-term trends of global FVOC emissions by developing a new global anthropogenic FVOC emission inventory (MEIC-global-FVOC). The FVOC emissions fill the missing components in traditional emission inventories, providing a more complete representation of long-term organic compound emissions. Importantly, the emission growth rates of L/SVOC and IVOC exceed those of POA and NMVOC, revealing the emerging importance of the FVOC emission inventory. Based on the new inventory, we track the dynamics and decompose the driving factors of global FVOC emissions across volatility bins and source sectors from 1970 to 2020, revealing distinct source contributions, volatility distributions, and growth patterns. In the future, additional data products, including emissions of individual chemical species and high-resolution emission grids, will be developed to support CTM simulations and health risk assessments. The MEIC-global-FVOC emission inventory presented in this study is expected to serve as a high-quality, open-access foundation for air pollution research and mitigation, advancing the understanding of the chemical, climate, and health impacts of atmospheric organic compounds.

## METHODS

### Model framework and emission estimate methods

The MEIC-global-FVOC emission inventory is built upon the MEIC-global-NMVOC emission inventory [27]. A five-level source category system, comprising major sectors, sectors, fuels/products/processes, technologies, and emission control measures, is used to represent anthropogenic FVOC emission sources and related technologies (Table S1), excluding open biomass burning, agriculture, and waste treatment. The inventory covers 228 countries or territories which are listed in Table S2 with their ISO-3166 country codes. Detailed descriptions of the source categories and the country/territory definition have been provided in Sections 2.1 and 2.2 of Xu *et al.* (2025) [27].

The MEIC-global-FVOC inventory categorizes emissions into 10 volatility bins (Table S3), including 2 LVOC bins, 3 SVOC bins, 4 IVOC bins, and 1 NMVOC bin that are defined by the logarithm of saturation vapor concentration (log_10_C*). Under this definition, the particle-phase fractions of L/S/IVOC emissions correspond to POA in traditional inventories. However, unlike the non-volatile POA typically assumed in those inventories, L/S/IVOC can undergo gas-particle partitioning as environmental conditions change (see Text S1 for details).

The country-level emissions for each volatility bin from 1970 to 2020 are estimated using the following equations:


(1) \begin{eqnarray*}
{E}_{i,v} = \mathop \sum \limits_j \mathop \sum \limits_k \left[ {{A}_{i,j,k} \times \left( {\mathop \sum \limits_m {X}_{i,j,k,m} \times E{F}_{i,j,k,m,v}} \right)} \right],
\end{eqnarray*}

(2) \begin{eqnarray*}
E{F}_{i,j,k,m,v} &=& E{F}_{{unabated},i,j,k,m,v}\\
&&\times \mathop \sum \limits_n \left[ {{C}_{i,j,k,m,n} \times \left( {1 - {\eta }_{v,n}} \right)} \right],
\end{eqnarray*}

where *i, j* and *v* represent country, sector, and volatility bin, respectively. *k* represents fuel type for combustion-related sources or product for process-related sources. *m* represents the technology applied in combustion, industrial production, or solvent use. *E* represents FVOC emissions, calculated from activity rate (*A*), technology distribution (*X*), and emission factor ($EF$). The emission factor is determined by integrating unabated emission factor ($E{F}_{{unabated}}$) with penetration ratios of control technologies (*C*) and their removal efficiencies ($\eta $). In particular, emissions from thermal power, iron and steel, and cement industries are considered as point sources, and transport emissions are calculated by fleet-based models. Detailed descriptions for point-source estimates and fleet-based models can be found in our previous work [27,28,50].

The methods for deriving activity rates, technology distributions, unabated NMVOC emission factors, NMVOC emission control measures, and NMVOC emission estimates are provided in Xu *et al.* (2025) [27]. Therefore, we focus on the methods for deriving L/S/IVOC emission factors and estimating L/S/IVOC emissions in this study.

### Emission factors

L/S/IVOC undergo gas-particle partitioning in the atmosphere. We therefore prioritize using unabated emission factors derived from experiments that measured both of gas- and particle-phase emissions (e.g. by TD-GC-MS and GC × GC-ToF-MS). When such data are unavailable, emission factors are either calculated by scaling from POA or NMVOC emission factors, or obtained directly from literature estimates (e.g. mass-balance approaches). About 82%, 88%, and 61% of global LVOC, SVOC, and IVOC emissions in 2020, respectively, are estimated by using measured emission factors. Regional variations in unabated emission factors are considered when available. The values of unabated emission factors and their data sources are summarized in Table S4.

To clarify the quality and relative importance of emission factors from different sources, a confidence level is assigned for each unabated emission factor based on its derivation (e.g. direct measurements locally or adoption from other regions; Table S4). The proportions of emission factors at different confidence levels are then evaluated for each region and major sector. The quality is generally higher in measurement-intensive regions, such as China and the U.S., and lowest in Latin America and Africa. Notably, most L/S/IVOC emissions are estimated using higher-quality emission factors (i.e. confidence levels A+, A, and B). A detailed description of emission factor quality evaluation is provided in Text S1.

#### Coal combustion

For residential coal combustion, we apply the average of measured gas- and particle-phase unabated emission factors from the experiments conducted in China [11,22,51], determined using both GC × GC-ToF-MS and PTR-ToF-MS. For the power and industry sectors, such data are unavailable, for which the estimated emission factors from literatures are used [11].

#### Oil combustion

For on-road transport, vehicle-category-, fuel-type-, and emission-standard-specific emission factors are obtained from the measurements in the U.S. [52,53] (analyzed using TD-GC-MS) and China [54–56] (analyzed using GC-MS/FID, GC × GC-ToF-MS, or TD-GC-MS). Total OC comparisons with the EC/OC analysis suggest about 50%–70% of L/SVOC are recovered by these measurements [52,53]. To correct for this, we assume that 30%, 50%, and 30% of L/SVOC are missing in the measured emission factors for gasoline vehicles, nonaftertreatment diesel vehicles, and diesel particulate filters (DPF)-equipped diesel vehicles, respectively, if not already corrected in the reported emission factors. For off-road machinery, emission factors are taken from the measurements in the U.S. [52,53] (analyzed using TD-GC-MS) and China [57,58] (analyzed using GC-MS/FID and TD-GC-MS). For oil combustion in the power, industry, and residential sectors, where direct measurements are unavailable, estimated emission factors from literatures are used.

#### Biofuel combustion

For residential biofuel combustion, we collect measured unabated emission factors from the experiments in the U.S. [24] (analyzed using TD-GC-MS), China [22,59] (analyzed using GC × GC-ToF-MS and PTR-ToF-MS), and India [23,60] (analyzed using DC-GC-FID, GC × GC-FID, PTR-ToF-MS, and GC × GC-ToF-MS), distinguishing between wood and crop combustion. The average emission factors for wood and crop combustion in China and India are applied to themselves and other developing countries in South and Southeast Asia, where both biofuel types are widely consumed [15]. Emission factors for wood combustion in the U.S. and China are applied to developed and other developing countries, respectively. The emission factors are further constrained by measured POA emission factors or field observations as described in Text S2.

#### Fugitive and industrial process

Given the limited availability of measurements, the unabated L/SVOC emission factors for fugitive and industrial process are calculated from OC emission factors, and the unabated IVOC emission factors are derived from NMVOC emission factors [27], as detailed in Text S2.

#### VCPs use

In addition to NMVOC, the use of VCPs emits IVOC and a small amount of SVOC. For industrial paint use, the total unabated S/IVOC emission factors are obtained from measurements [61], analyzed using TD-GC × GC-ToF-MS and Vocus PTR-ToF. For other use types, the total unabated S/IVOC emission factors are derived from mass-balance-based estimates that combine organic solvent content, S/IVOC profiles, and volatilization fractions of different VCPs [31]. These emission factors are then allocated to each volatility bin by applying measured volatility distributions [62] and inferred distributions from US EPA’s VCPy framework [36]. For domestic use of VCPs, per capita S/IVOC emission factors are applied and are assumed to scale with country-specific per capita NMVOC emission factors [27].

Because of limited availability of direct measurements and technical documentation, control technologies and removal efficiencies for L/SVOC are assumed to be equivalent to those of PM_2.5_, and IVOC emission control is assumed to follow that of NMVOC. The penetration of NMVOC control technologies is modeled by a policy-driven technology turnover model, as described by Xu *et al.* (2025) [27]. PM_2.5_ control technology penetration is either modeled using a similar turnover model or obtained from the Global Infrastructure emission Detector (GID, http://gidmodel.org.cn/). For transport sources, the emission factors are integrated within the fleet-based models that synchronously account for unabated emission factors and control measures; thus, the penetration of control technologies is not estimated separately. Detailed descriptions of emission factor selection and treatments are presented in Text S2.

## Supplementary Material

nwag281_Supplemental_Files
