# Supplementary material for: Global emission trends of anthropogenic full-volatility-range organic compounds 1970–2020
Source: Natl Sci Rev. 2026 May 18;13(11):nwag281. doi: 10.1093/nsr/nwag281 (PMC13289744; doi:10.1093/nsr/nwag281)
Supplement: nwag281_Supplemental_Files [file nwag281_supplemental_files.zip › 2.SI.pdf]

Supplementary Information of

Global emission trends of anthropogenic full-volatility-range organic compounds 1970-2020

Ruochong Xu[1], Ruqian Miao[2], Jingxian Li[3], Hanchen Ma[1], Hanwen Hu[3], Huaxuan Wang[1], Xizhe Yan[1], Xiaodong Liu[3], Dan Tong[1], Guannan Geng[3], Qi Chen[2], Kebin He[3,4], and Qiang Zhang[1,*]

[1]Department of Earth System Science, Ministry of Education Key Laboratory for Earth System Modeling, Institute for Global Change Studies, Tsinghua University, Beijing 100084, People's Republic of China
[2]State Key Laboratory of Regional Environment and Sustainability, College of Environmental Sciences and Engineering, Peking University, Beijing, 100871, China
[3]State Key Laboratory of Regional Environment and Sustainability, School of Environment, Tsinghua University, Beijing 100084, China
[4]Institute for Carbon Neutrality, Tsinghua University, Beijing 100084, People's Republic of China

*Corresponding author: Qiang Zhang (qiangzhang@tsinghua.edu.cn)

Supplementary Text S1. Uncertainty analysis and limitations

Uncertainty analysis. In this study, uncertainties of emission estimates are quantified by a Monte Carlo approach, following the methods in our previous work [1,2]. The term "uncertainty" here refers to the 95% confidential interval (CI) around the central estimates. The probability distributions of emission parameters are presented in Table S5. The uncertainty analysis of NMVOC emissions is provided in our previous work [2].

Globally, the uncertainties of L/SVOC emissions range from -67% to +76% in 1970 and from -53% to +61% in 2020, while those of IVOC emissions range from -62% to +82% in 1970 and from -50% to +69% in 2020 (Table S6). The uncertainty ranges have narrowed over time, due to improvements in statistical reporting systems for activity rates and the increased availability of information for other parameters. The upper bounds of the uncertainty ranges tend to deviate more from the central estimates than the lower bounds, indicating a larger likelihood of underestimation. This is likely due to the difficulties in fully capturing L/S/IVOC during emission factor measurements and aligns with the underestimation of OA commonly found in CTM simulations [3-5]. The uncertainties of regional L/S/IVOC emissions vary (Table S6), depending on the quality of activity rates, the availability of locally measured emission factors, and the relative contributions from source categories with different uncertainty levels.

This study has limitations in different aspects as discussed below.

Source categories. Emissions from open biomass burning and waste treatment are not included, which may be important in certain regions. These sources will be incorporated in future versions of MEIC-global-FVOC.

POA definition. In the full-volatility-range framework, the particle-phase fractions of L/S/IVOC emissions correspond to POA in traditional inventories. However, unlike the non-volatile POA typically assumed in those inventories, L/S/IVOC can undergo gas-particle partitioning as environmental conditions change. This provides a more detailed representation of the volatility distribution of organic compounds, which is important for CTM simulations.

The POA definition should be carefully considered in specific analyses and comparisons. For example, in some L/S/IVOC emission factor measurements, comparison between total organic carbon and EC/OC analysis suggest that only about 50%-70% of L/SVOC are recovered [11,12], indicating that not all POA, as traditionally defined, are captured. In such cases, emission factor corrections are applied as described in the Methods section and Supplementary Text S2. In addition, when comparing with traditional POA emission inventories, the environmental conditions under which the POA emission factors used are measured are often unknown (i.e., what proportions of particle-phase L/S/IVOC are captured). This introduces additional uncertainties in emission comparisons, as detailed in the Discussion section. Importantly, within the current full-volatility-range framework, "non-volatile POA" is no longer assumed, and organic compounds are allowed to dynamically partition between the gas and particle phases. Therefore, environmental conditions and measurement techniques should be more carefully considered in relevant analyses.

For highly refractory organic aerosols, the correspondence to L/S/IVOC are difficult to define within the framework of this study. Highly refractory organic aerosols are defined based on thermal stability, whereas L/S/IVOC are classified by volatility, and these two properties are not directly comparable.

Some highly-refractory organic aerosols are likely LVOC because they do not evaporate even at elevated temperatures. Meanwhile, some LVOC species may decompose rather than evaporate at high temperatures and therefore may not appear as highly refractory. Establishing a quantitative definition is beyond the scope of this study.

Emission factors.

To clarify the quality and relative importance of emission factors from different sources, a confidence level is assigned for each unabated emission factor based the criteria below, as shown in Table S4.

- **A+:** EFs measured locally;
- **A:** EFs measured in other regions but constrained by locally measured POA EFs or field observations;
- **B:** EFs measured in other regions without any constraints, or EFs derived by mass-balance-based estimates;
- **C:** EFs calculated from OC and NMVOC EFs, constrained by OC EFs and source profiles;
- **D:** EFs lacking available measurements or mass-balance-based estimates and taken from estimated values in literatures.

The proportions of emission factors at different confidence levels are evaluated by number and emission contribution for each region and major sector, as shown in Table S11. The quality of emission factors is generally higher in measurement-intensive regions, such as China and the U.S., and are lowest in regions like Latin America. For example, nearly 60% of emission factors for oil combustion in China and Canada and the U.S. are classified as A+ by number, whereas about 65% are classified as B in Latin America and Africa. Notably, most L/S/IVOC emissions are estimated using higher-quality emission factors (A+, A, and B). For example, in most regions, over 70% of L/S/IVOC emissions from coal and biofuel combustion are estimated using emission factors with confidence levels of A+, A, and B. This supports the reliability of emission estimates, given current measurement availability. However, emission factors with confidence levels C and D still account for non-negligible proportions in some sectors, such as oil combustion. For fugitive and industrial process, almost all emission factors are derived by scaling from POA or NMVOC emission factors (confidence level C). This highlights the need for full-volatility-range emission factor measurements across a wider variety of emission sources.

As discussed above, the availability of measured gas- and particle-phase emission factors remains limited, and substantial discrepancies are sometimes reported for the same sources, making emission estimates highly sensitive to the choice of emission factors. In regions lacking local measurements, emission factors adopted from other regions may not be sufficiently representative of local conditions. For example, stove and fuel types used for residential combustion in Africa can differ markedly from those in China and India, where most measurements have been conducted; the composition and formulation of VCPs may also vary substantially across regions. More careful evaluation is needed when analyzing emission trends and simulating OA in CTMs in these regions without local measurements. In addition, some emission factors are indirectly derived from POA or NMVOC data, or constrained by observations (Fig. S11), which may introduce additional uncertainties or biases. Future versions of MEIC-global-FVOC will incorporate more locally

measured emission factors, as such data become available, to further improve emission estimates.

Supplementary Text S2. Emission factors

Coal combustion

For residential coal combustion, we apply the average of measured gas- and particle-phase unabated emission factors from the experiments conducted in China [6-8], determined using both GCxGC-ToF-MS and PTR-ToF-MS. For the power and industry sectors, such data are unavailable, for which the estimated emission factors from literatures are used [6]. Country-specific differences are not considered due to limited data availability.

Because residential fuel combustion generally lacks end-of-pipe control, the unabated emission factors are treated as final emission factors. For the power and industry sectors, unabated IVOC emission factors are used directly, considering that NMVOC control is rarely implemented [2]. However, emission control is considered for L/SVOC emission factors of power and industry sectors, reflecting the widespread implementation of $PM_{2.5}$ abatement technologies.

For coal combustion in thermal power, iron and steel, and cement industries treated as point sources, control technologies and removal efficiencies are obtained from the GID platform. In GID, unit-level control information is directly collected from regional datasets (e.g., EPA's eGRID) when available; otherwise, it is modeled by a unit-level turnover algorithm to represent the compliance with increasingly stringent $PM_{2.5}$ emission standards, prioritizing the installation of control technologies on large and newly-built units. Details about the GID methodology are provided in our previous work [1,9,10]. For coal combustion in other industries, $PM_{2.5}$ emission control is modeled at country level using a policy-driven technology turnover model. A set of $PM_{2.5}$ emission standards are compiled from regional policies on industrial boilers (see Table S7), and the control technologies/removal efficiencies required to meet those standards are then assessed. The time series of control technology penetration is modeled by S-shaped curves, driven by the implementation and upgrading of emission standards, as described in our previous work [2].

Oil combustion

The transport sector is the major source of oil-combustion emissions. For on-road transport, vehicle-category-, fuel-type-, and emission-standard-specific emission factors are obtained from the gas- and particle-phase measurements in the U.S. [11,12] (analyzed using TD-GC-MS) and China [13-15] (analyzed using GC-MS/FID, GC×GC-ToF-MS, or TD-GC- MS). Total organic carbon (OC) comparisons with the EC/OC analysis suggest about 50%-70% of L/SVOC are recovered by these measurements (e.g., by using gas chromatography-mass spectrometers) [11,12]. To correct for this, we assume that 30%, 50%, and 30% of L/SVOC are missing in the measured emission factors for gasoline vehicles, nonaftertreatment diesel vehicles, and diesel particulate filters (DPF)-equipped diesel vehicles, respectively, if not already corrected in the reported emission factors. For vehicle categories or emission standards that are not covered by the measurements, data from vehicles with similar load capacity and the closest emission standard are used. We apply the emission factors measured in the U.S. and China to developed and developing countries, respectively. An exception is made for Europe where the emission factors reported for Chinese vehicles are used due to the alignment of emission standard systems in the two regions [16].

For off-road machinery, emission factors are taken from the gas- and particle-phase measurements in the U.S. [11,12] (analyzed using TD-GC-MS) and China [17,18] (analyzed using GC-MS/FID and TD-GC-MS). Emission standards are not considered for off-road machinery. Similar to the treatment for on-road vehicles, corrections are applied to L/SVOC emission factors, and the U.S. and Chinese data are assigned to developed and developing countries, respectively. In addition, emission factors for navigation and aviation are derived from measurements and reported values in previous studies, respectively [19,20].

For oil combustion in the power, industry, and residential sectors, where direct measurements are unavailable, estimated emission factors from literatures are used without considering country-specific differences [6]. Emission control treatments follow the same approach as for coal combustion (Sect. *Coal combustion*).

Biofuel combustion

For residential biofuel combustion, we collect measured gas- and particle-phase unabated emission factors from the experiments in the U.S. [21] (analyzed using TD-GC-MS), China [22,23] (analyzed using GC×GC-ToF-MS and PTR-ToF-MS), and India [24,25] (analyzed using DC-GC-FID, GC×GC-FID, PTR-ToF-MS, and GC×GC-ToF-MS), distinguishing between wood and crop combustion. The average emission factors for wood and crop combustion in China and India are applied to themselves and other developing countries in South and Southeast Asia, where both biofuel types are widely consumed [26]. Emission factors for wood combustion in the U.S. and China are applied to developed and other developing countries, respectively.

Given that residential biofuel combustion is the major anthropogenic source of POA [26-28], we evaluate the collected data by calculating particle-phase L/SVOC emission factors ($EF_{particle}$) using equation (3) [29] and comparing them with the POA emission factors (derived from OC emission factors):

$$EF_{particle} = \sum_{v} \left[EF_v \times \left(1 + \frac{C_v^*}{C_{OA}}\right)^{-1} \right] \quad (3)$$

where C^* and C_{OA} represent saturation vapor concentration and organic aerosol concentration at the emission outlet, respectively. C_{OA} is assumed to be 10^4 μg/m^3. Other symbols are as defined in equation (1).

The particle-phase L/SVOC emission factors are comparable with locally measured POA emission factors in the U.S., but lower than those in developing regions such as China, India, and Africa. This may result from incomplete recovery of L/SVOC species during measurements or regional differences in stove types and combustion conditions [21,22]. To avoid underestimation, L/SVOC emission factors in these regions are constrained using equation (4):

$$EF_{constr,v} = EF_v \times \frac{EF_{POA}}{EF_{particle}} = EF_v \times \frac{EF_{OC} \times R_{OMtoOC}}{EF_{particle}} \quad (4)$$

where EF_{constr}, EF_{POA}, and EF_{OC} denote the constrained L/SVOC emission factors, POA emission factors, and locally measured OC emission factors, respectively. R_{OMtoOC} represents the organic matter (OM) to OC mass ratio, which is assumed to be 1.4. The regional values and sources of EF_{OC} are summarized in Table S8. As shown in our companion paper, this constraint improves

the agreement between observed and modeled OA concentrations in these regions [4].

In Europe, the modeled POA is substantially lower than the derived POA concentrations by positive matrix factorization (PMF) on aerosol mass spectrometer (AMS) data [4,30]. We attribute the biases to the underestimation of biomass-burning-related OA (BBOA) because BBOA in Europe are mostly contributed by residential biofuel combustion that are often not well characterized [28,31,32]. It is difficult to determine whether the biases are caused by underrepresented activity rates (e.g., from underreported rural biofuel consumption) or potentially lower emission factors when the U.S.-based emission measurements do not represent the European conditions. We therefore constrain the L/SVOC emission factors in Europe as below:

$$EF_{constr,v} = EF_v \times \frac{BBOA_{obs}}{BBOA_{sim}} \quad (5)$$

where $BBOA_{obs}$ and $BBOA_{sim}$ denote the regional average concentrations of observed and simulated BBOA, respectively. We treat Europe as four sub-regions (i.e., Western, Eastern, Northern, and Southern Europe) for calculating the regional observed-to-simulated BBOA ratios (Table S9). Details on the OA modeling and comparisons are presented in our companion paper [4].

For biofuel combustion in the power and industry sectors, where measurements are unavailable, we use the estimated unabated emission factors from literatures [6,33]. Emission control treatments follow the same approach as for coal combustion (Sect. *Coal combustion*).

Fugitive and industrial process

Given the limited availability of measurements, the unabated L/SVOC emission factors for fugitive and industrial process are calculated from OC emission factors using equation (6), assuming the volatility distributions are analogous to that of coal combustion [6]:

$$EF_{i,j,k,m,v} = EF_{OC,i,j,k,m} \times R_{OMtoOC} \times \frac{EF_{coal,v}}{\sum_{L/SVOC} EF_{coal,v}} \quad (6)$$

where EF_{coal} represents the unabated L/SVOC emission factor for coal combustion. Other symbols are as defined in equation (1) and (4). The values and sources of EF_{OC} are summarized in Table S8. The unabated IVOC emission factors for fugitive and industrial process are derived from NMVOC emission factors using equation (7).

$$EF_{i,j,k,m,v} = EF_{NMVOC,i,j,k,m} \times \frac{M_{j,k,v}}{M_{j,k,NMVOC}} \quad (7)$$

where EF_{NMVOC} represents the unabated NMVOC emission factor, and M denotes the mass fraction of each volatility bin. NMVOC emission factors are provided by Xu et al. (2025) [2]. Mass fractions are obtained from available measurements [34]; otherwise, the source profiles from the SPECIATE v5.2 database [35] (available at https://www.epa.gov/air-emissions-modeling/speciate-2) are used to calculate the volatility-bin-specific mass fractions, based on the saturation vapor pressure of individual chemical species from the OPEn structure–activity/property Relationship App (OPERA) [36].

The NMVOC control technology penetration and removal efficiencies from Xu et al. (2025) [2] are applied to the IVOC emission factors for fugitive and industrial process. For L/SVOC emission

factors, the emission control for iron and steel, cement, and other industries, as derived in Sect. *Coal combustion*, are applied according to the source mapping in Table S10.

VCPs use

In addition to NMVOC, the use of VCPs emits IVOC and a small amount of SVOC. For industrial paint use, the total unabated S/IVOC emission factors are obtained from measurements [37], analyzed using TD-GC×GC-ToF-MS and Vocus PTR-ToF and distinguishing between water- and solvent-borne paints. For other use types, the total unabated S/IVOC emission factors are derived from mass-balance-based estimates that combine organic solvent content, S/IVOC profiles, and volatilization fractions of different VCPs [38]. These emission factors are then allocated to each volatility bin by applying measured volatility distributions [39] and inferred distributions from U.S. EPA's VCPy framework [40]. For domestic use of VCPs, per capita S/IVOC emission factors are applied and are assumed to scale with per capita NMVOC emission factors, which vary by country due to differences in income levels and environmental conditions, as described in Sect. 2.4.6 of Xu et al. (2025) [2]. The NMVOC emission control from Xu et al. (2025) [2] is applied to the IVOC emission factors for VCPs use.

Other sources

For the remaining sources, including the combustion of natural gas, coke oven gas, blast furnace gas, and other fuels (e.g., liquefied petroleum gas, LPG), unabated emission factors are obtained from estimated literature values [6,33]. No emission control is considered for these sources.

Supplementary Text S3. Sensitivity analysis

The year of 2020. Emissions in 2020 were substantially affected by the COVID-19 pandemic, leading to atypical reductions in certain sources. To assess whether including 2020 influences the trend analysis, we compare emission changes and growth rates over 1970-2020 and 1970-2019 (see Tables S12 and S13). When 2020 is excluded (i.e., 1970-2019), emission growth is slightly stronger than that over 1970-2020. However, the magnitudes of emission changes and growth rates remain close (e.g., differences in average growth rate are within ±1.5% per decade globally and in most regions).

Importantly, including 2020 does not affect the key comparative results. For example, an important comparative result is that the emission growth rates of L/SVOC and IVOC (9.7% and 11.5% per decade during 1970-2020) exceed that of NMVOC (8.4% per decade), leading to a widening gap between full-volatility-range and traditional emission inventories over time. When 2020 is excluded, global emission growth rates of L/SVOC, IVOC, and NMVOC increase to +10.3%, +12.5%, and +9.8% per decade, and the overall comparative conclusion remains unchanged.

Volatility bin definition. FVOC emission are categorized into 10 volatility bins based on $\log_{10}C^*$ (Table S3). However, C^* depends on environmental conditions, which may vary among the emission factor measurements. For example, the C^* of a specific chemical species can vary by one or more orders of magnitude across the range of ambient temperatures [50]. We therefore conduct a sensitivity analysis to assess whether shifts in defined volatility-bin boundaries would alter the reported trends. Three cases are considered:

- ***Base***: The definitions in Table S3, as used throughout the analysis in the Main Text.
- ***Higher***: The volatility bins are shifted by one bin toward higher volatility. The $\log_{10}C^*$ bins of ≤-2 to 3 represent L/SVOC. Because a detailed volatility distribution of NMVOC emissions ($\log_{10}C^*$ bin of > 7) is unavailable, we conservatively assume that 10% of emissions in the $\log_{10}C^*$ bins of > 7 belong to IVOC. Accordingly, the $\log_{10}C^*$ bins of 4 to 6, together with 10% of emissions in the $\log_{10}C^*$ bins of > 7, represent IVOC, and the remaining 90% of emissions in the $\log_{10}C^*$ bins of > 7 represent NMVOC.
- ***Lower:*** The volatility bins are shifted by one bin toward lower volatility. The $\log_{10}C^*$ bins of ≤-2 to 1 represent L/SVOC, the $\log_{10}C^*$ bins of 2 to 5 represent L/SVOC, and the $\log_{10}C^*$ bins of 6 to > 7 represent NMVOC.

Global emission changes and growth rates of L/SVOC, IVOC, and NMVOC are presented in Table S14. The results show that when the volatility bins are shifted by one bin, differences in emission changes during 1970-2020 are generally within ±4%, and differences in growth rates are mostly within ±1%. Also, the growth rates of L/SVOC and IVOC emissions still exceed that of NMVOC emissions. Therefore, the major analysis and conclusions of this study are not sensitive to moderate shifts in the defined volatility-bin boundaries.

Control efficiencies. In this study, the control efficiencies for L/SVOC and IVOC are assumed to be equivalent to those of $PM_{2.5}$ and NMVOC, given limited availability of direct measurements and

technical documentation. This assumption may not fully capture the effects of control technologies or environmental conditions at emission outlets. For example, SVOC may evaporate during dilution and thus may not be fully captured by $PM_{2.5}$ control technologies, while IVOC may not be efficiently removed by regenerative thermal oxidizers (RTO), potentially leading to an overestimation of control efficiencies. In contrast, IVOC may be more easily adsorbed by activated carbon, or water-soluble SVOC may be more efficiently removed by wet scrubbers, potentially resulting in an underestimation of control efficiencies. We therefore conduct a sensitivity analysis covering a wide range of control efficiencies to assess whether this assumption would alter the reported trends and comparative results. Three cases are considered:

- ***Base***: The control efficiencies for L/SVOC and IVOC are assumed to be equivalent to those of $PM_{2.5}$ and NMVOC, as used throughout the analysis in the Main Text.
- ***η_High***: The control efficiencies for L/SVOC and IVOC are 50% higher than those of $PM_{2.5}$ and NMVOC, respectively (not exceeding 100%).
- ***η_Low:*** The control efficiencies for L/SVOC and IVOC are 50% lower than those of $PM_{2.5}$ and NMVOC, respectively.

Global emission changes and growth rates of L/SVOC and IVOC under the three cases are presented in Table S15. The results show that when control efficiencies become higher, emission increases and growth rates become slightly lower but remain close to the *Base* case. However, when control efficiencies are substantially lower, emission increases and growth rates become noticeably higher, especially for L/SVOC emissions. Nevertheless, the overall trends and magnitudes remain largely comparative to the *Base* case. In any case, the growth rates of L/SVOC and IVOC emissions still exceed that of NMVOC emissions, which is even more pronounced under the *η_Low* case. Considering that the ±50% variation in control efficiencies represents a substantial change and that the actual differences are likely much smaller in reality, we conclude that the major analysis and conclusions regarding emission growth would not be substantially affected by the assumed control efficiencies.

Regional grouping. In this study, countries/territories worldwide are grouped into nine regions (i.e., China, India, Rest of Asia, Africa, Canada and the U.S., Latin America, Western Europe, Eastern Europe, and Oceanic and Pacific), based on both the level of economic development and geographical proximity. Similar levels of economic development imply more comparable affordability of consumer products and adoption of production/emission-control technologies, while geographical proximity indicates more similar environmental conditions (e.g., available fuel types, heating demand, temperature-dependent evaporation) that may influence emission intensities.

Within these regions, China, India, and Africa represent developing regions with high emission contributions (e.g., 22%, 12%, and 20% of global FVOC emissions in 2020) and substantial emission growth in 1970-2020. Canada and the U.S., Western Europe, and Eastern Europe represent developed regions, characterized by high contributions in the early years (e.g., 27%, 14%, and 9% of global FVOC emissions in 1970) and stable or declining trends. These regional definitions are commonly adopted in previous studies [1,2,9,27,33]. The remaining countries/territories are grouped into Rest of Asia, Latin America, and Oceanic and Pacific. This grouping achieves a relatively balanced distributions of emissions among regions (with comparable magnitudes, except

for Oceanic and Pacific) while capturing key regional features, such as increasing or decreasing trends and differences in volatility distributions (Figs. 4 and S4).

The regional grouping does not influence the analysis and main conclusions at global scale. Moreover, because the dominant regional emission contributors with distinct features are explicitly represented, most regional-scale analysis and conclusions would not be sensitive to minor or moderate adjustments in regional definitions (e.g., grouping China with East Asia, combining India with South and Southeast Asia, or distinguishing OECD countries).

Supplementary Text S4. Validation by GEOS-Chem simulations

Model configuration. We apply the GEOS-Chem chemical transport model (version 14.3.0, DOI: 10.5281/zenodo.10640536) to evaluate the robustness of MEIC-global-FV by comparing simulated results with observations. Global simulations are performed at 2°×2.5° horizontal resolution with 47 vertical levels for the year of 2018, driven by MERRA-2 reanalysis meteorological fields, with a one-month spin-up period. The model implements ozone-NO_x-hydrocarbon-aerosol-halogen chemistry in the troposphere [51]. Both gases and aerosols undergo dry deposition, with rates calculated online using a resistance in-series algorithm [52,53]. Wet deposition is calculated for water-soluble aerosols and gases [54]. A revised OA scheme, developed based on the ComplexSOA scheme in GEOS-Chem, is used to simulate OA, as described in our companion paper [4].

For air pollutants other than organic precursors, anthropogenic emissions are obtained from MEIC for China [55] and from CEDS for the rest of world [27]. Open biomass burning emissions are adopted from the Fire Inventory from the National Center for Atmospheric Research (NCAR) version 2.5 (FINNv2.5) [56]. Biogenic emissions are provided by the Model of Emissions of Gases and Aerosols from Nature (MEGAN v2.1) [57].

For anthropogenic organic precursors, the MEIC-global-FV emission inventory is used as input. For L/S/IVOC, volatility-bin-resolved emissions based on $\log_{10}C^*$ are used, consistent with the definitions in Table S3 and the precursor species in the revised OA scheme. For NMVOC, emissions are transformed to GEOS-Chem mechanism species using mapping tables developed by Carter (2023) and Li et al. (2014) [58,59]. Emissions are allocated into 0.1°×0.1° grids based on spatial surrogates: industry and commercial emissions are allocated by urban population [60-62]; rural and urban residential emissions are allocated by rural and urban population; aviation emissions are allocated by flight routes and airport locations [63]; railway emissions are allocated by railway network [64]; navigation emissions are allocated by shipping routes [65,66]; road transport emissions are allocated by vehicle-category- and road-type-dependent roadmaps [64]; the emissions from industrial process and solvent use are allocated by urban population; the emissions from other sources are allocated by population. Details of model configuration and the OA scheme are presented in our companion paper [4].

Ambient observations. Annual mean concentrations of OC in $PM_{2.5}$ are obtained from monitor networks, including the Interagency Monitoring of Protected Visual Environments (IMPROVE), the Chemical Speciation Network (CSN), the European Monitoring and Evaluation Programme (EMEP), and the China National Environmental Monitoring Centre (CNEMC), and the International Network to study Deposition and Atmospheric composition in Africa (INDAAF). 58 additional year-long measurements are compiled from literatures. For each data source, data from years closet to 2018 are used.

OA measurements by Aerodyne aerosol mass spectrometers (AMS) and aerosol chemical speciation monitors (ACSM) are obtained from literatures and the sites of the Aerosol, Clouds and Trace Gases Research Infrastructure (ACTRIS) network. Among the AMS and ACSM factors, hydrocarbon-like (HOA), biomass-burning-related (BBOA), coal-combustion-related OA factors (CCOA), and other primary factors are summed to represent observation-derived POA, while oxygenated OA (OOA) factors are summed to represent SOA. Details of the observational datasets and data processing are presented in our companion paper [4].

Comparison of observations and simulations. Using MEIC-global-FV as emission input, there is generally good agreement between observed and simulated OC, POA, and SOA concentrations (Fig. S12 and Table S16). The agreement is better under low or moderate concentration conditions, whereas under high concentrations (e.g., >10 μg/m^3 for POA and >15 μg/m^3 for SOA), simulations tend to underestimate observations. This discrepancy may result from some sub-grid sources or processes (e.g., wildfires or cooking) that are not resolved at the 2°×2.5° model resolution. In addition, heterogenous or liquid-phase reactions, which can be important under heavily polluted conditions but are underrepresented in GEOS-Chem, may also contribute to the underestimation, particularly for SOA [67].

Overall, the MEIC-global-FV emission inventory enables GEOS-Chem to reproduce not only total OA but also the relationships between primary and secondary components, outperforming many previous studies [3,5,30,31,68,69]. This provides support for the improvements and robustness of the emission estimates.

To further evaluate the improvements of MEIC-global-FV compared to existing inventories, two additional simulations are conducted:

- ***CEDS***: CEDS represents one of the most widely-used traditional global emission inventories. OC and NMVOC emissions from CEDS are used as input to GEOS-Chem, while other model configuration and emission inputs remain unchanged.
- ***CEDS+IVOC***: Based on the *CEDS* simulation, default IVOC emissions implemented in the GEOS-Chem ComplexSOA scheme are used [68,69], with all other configuration unchanged.

Figure S13 and Table S16 present the comparison of observed and simulated OC, POA, and SOA concentrations for the *CEDS* and *CEDS+IVOC* simulations. The results show that the simulation driven by MEIC-global-FV generally exhibits better overall model performance, although the *CEDS* and *CEDS+IVOC* simulations reproduce POA concentrations slightly better. The largest differences are found in SOA, where the *CEDS* and *CEDS+IVOC* simulations show substantial underestimation. These discrepancies are likely associated with the representation of L/SVOC emissions in MEIC-global-FV, which can partition between gas and particle phases and thus provide additional SOA precursors, as well as more accurate estimates of IVOC emissions. Overall, these results support the improvements of MEIC-global-FV, and highlights the importance of full-volatility-range emission input for OA modeling. As secondary pollution becomes increasingly important in many regions of the world [30,38,67], a full-volatility-range modeling framework is essential for understanding OA formation and designing effective mitigation strategies [4].

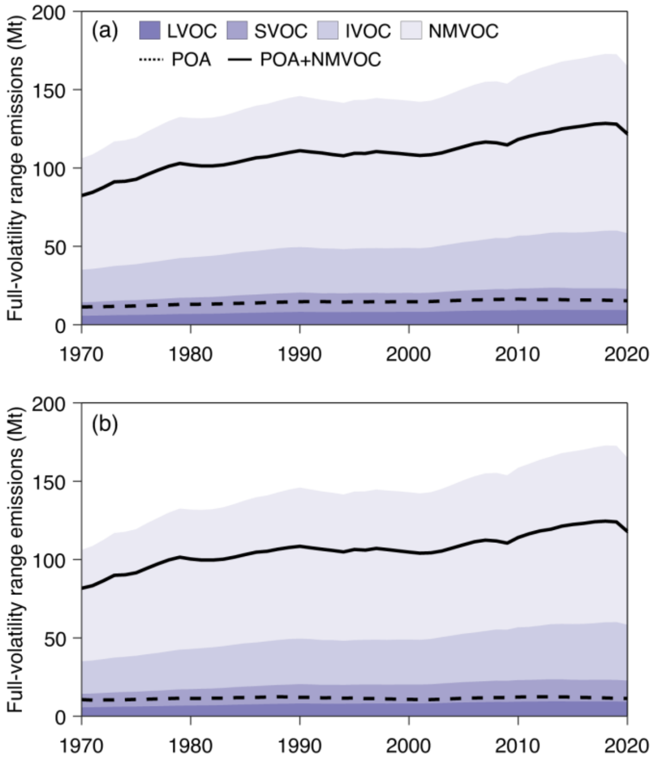

Figure S1. Global FVOC emission trends from 1970 to 2020, aggregated by volatility bin (LVOC, SVOC, IVOC, and NMVOC). The black dashed and solid lines represent global POA emissions and the sum of global POA and NMVOC emissions, respectively. The OC emissions are obtained from (a) CEDS and (b) EDGAR, respectively, and are converted to POA using an OM-to-OC ratio of 1.4.

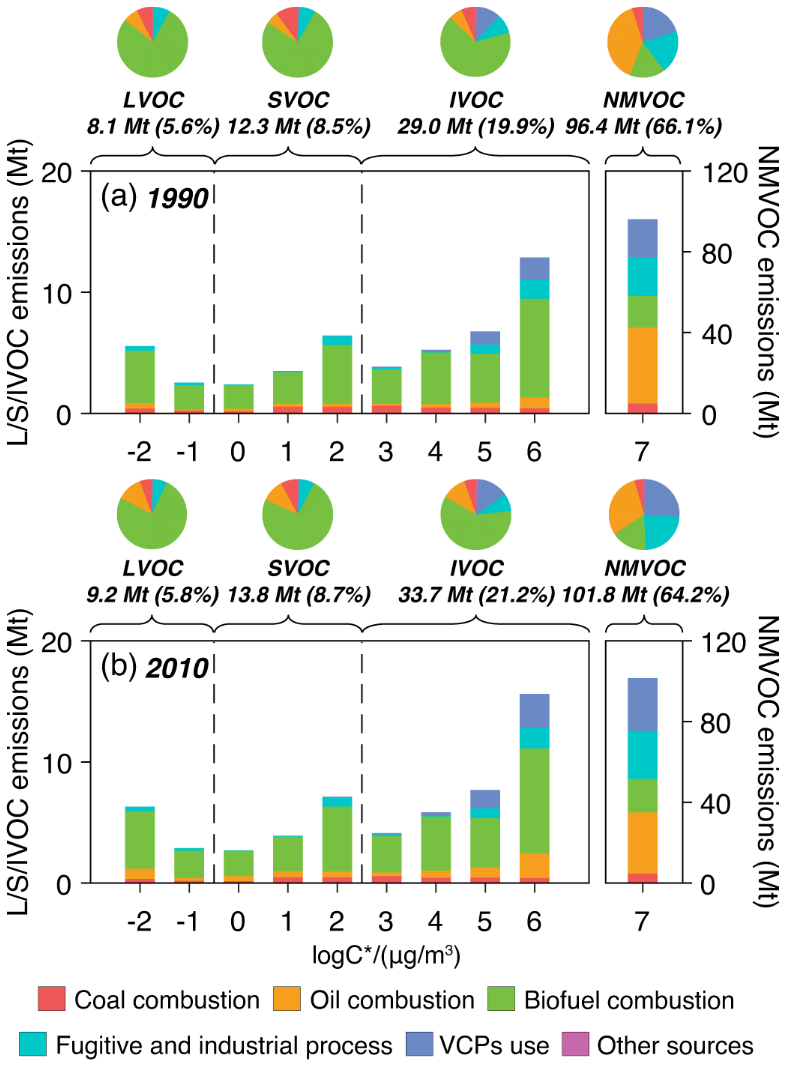

Figure S2. The same as Fig. 2a and 2b but for 1990 and 2010.

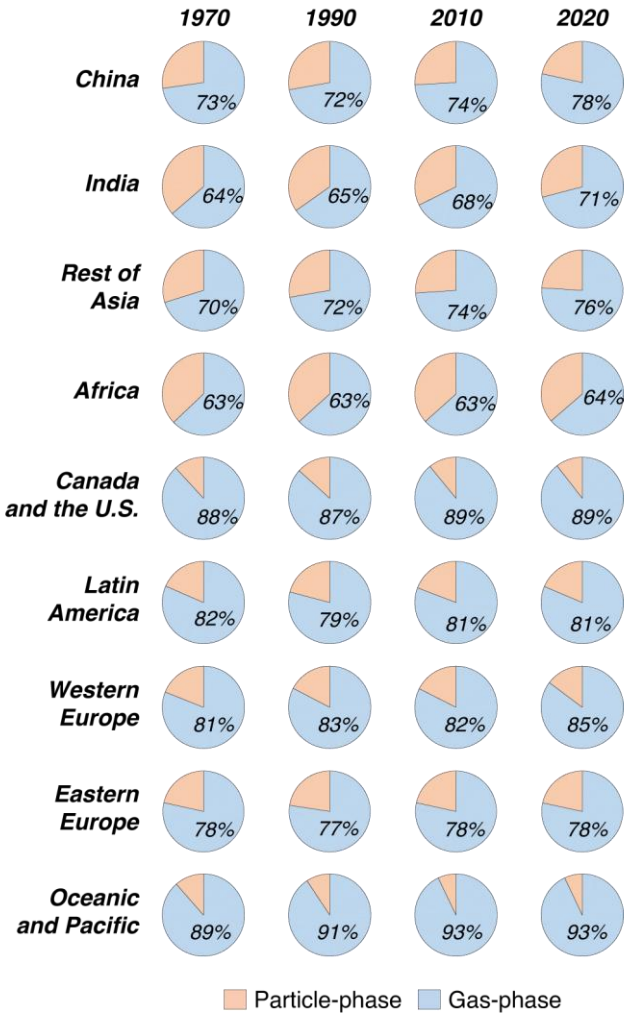

Figure S3. The gas- and particle-phase proportions of regional L/S/IVOC emissions in 1970, 1990, 2010, and 2020. Particle-phase emissions were estimated using the approach in Donahue et al. (2006) [29], and regional OA concentrations (C_{OA}) were taken from Huang et al. (2023) [33].

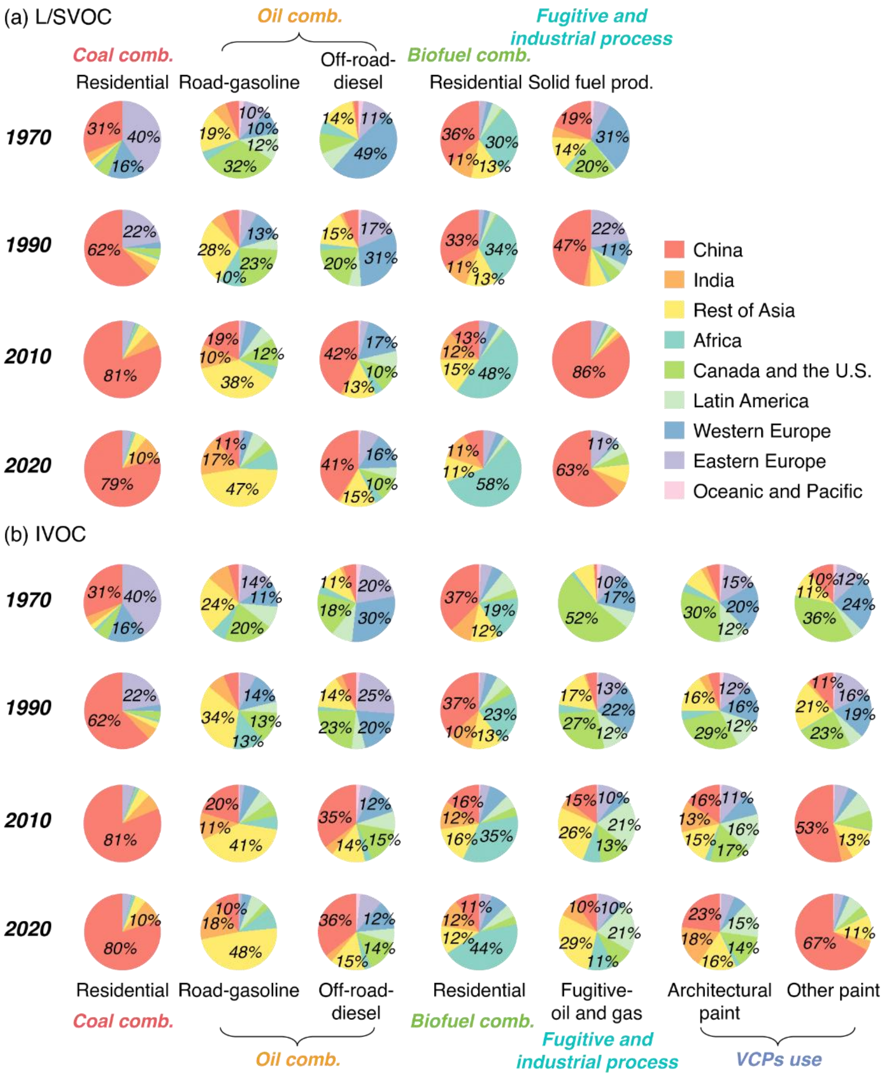

Figure S4. Regional contributions to (a) L/SVOC and (b) IVOC emissions in 1970, 1990, 2010, and 2020 for the selected main sources for each of the major sectors.

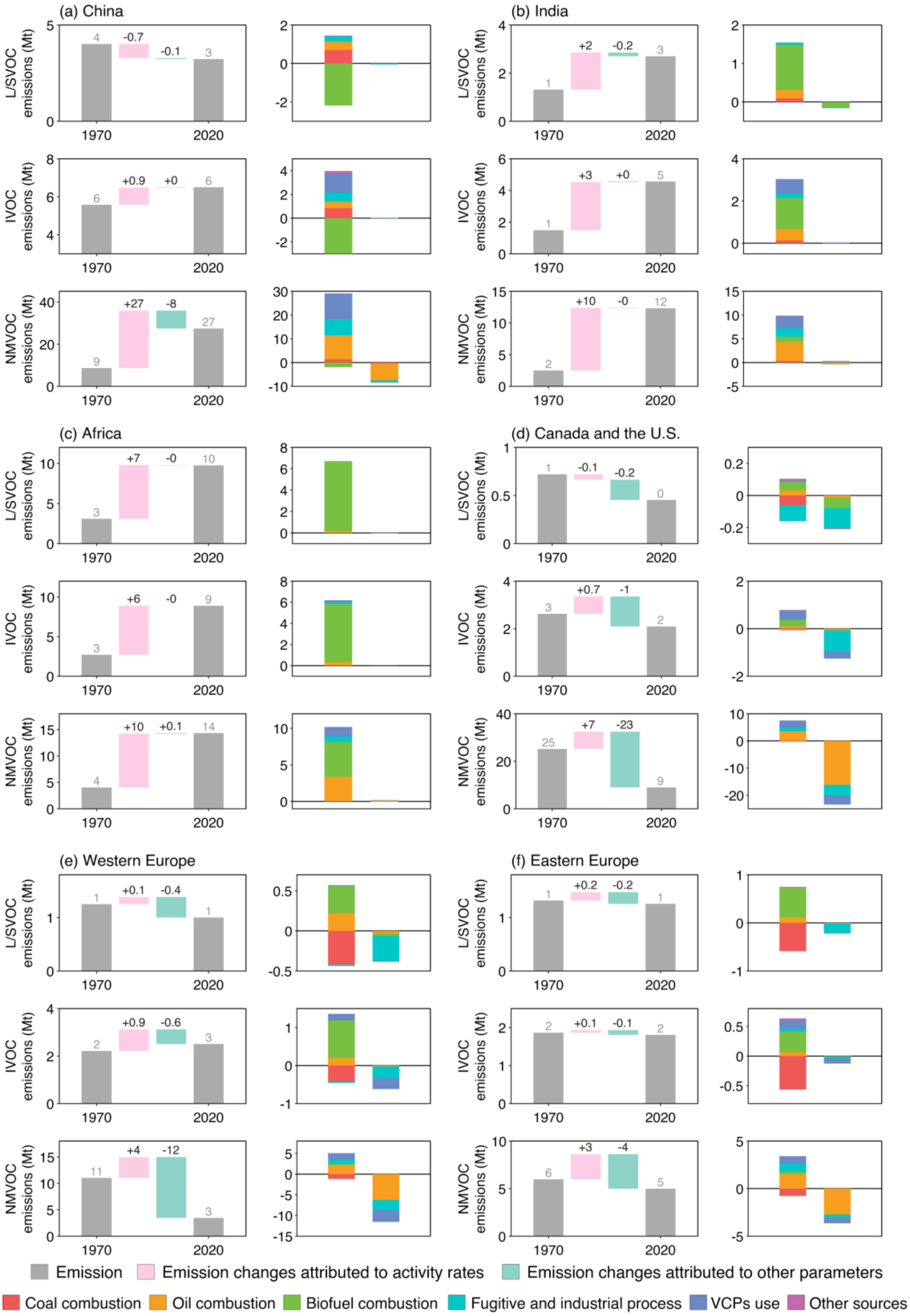

Figure S5. The same as Fig. 5 but for different regions.

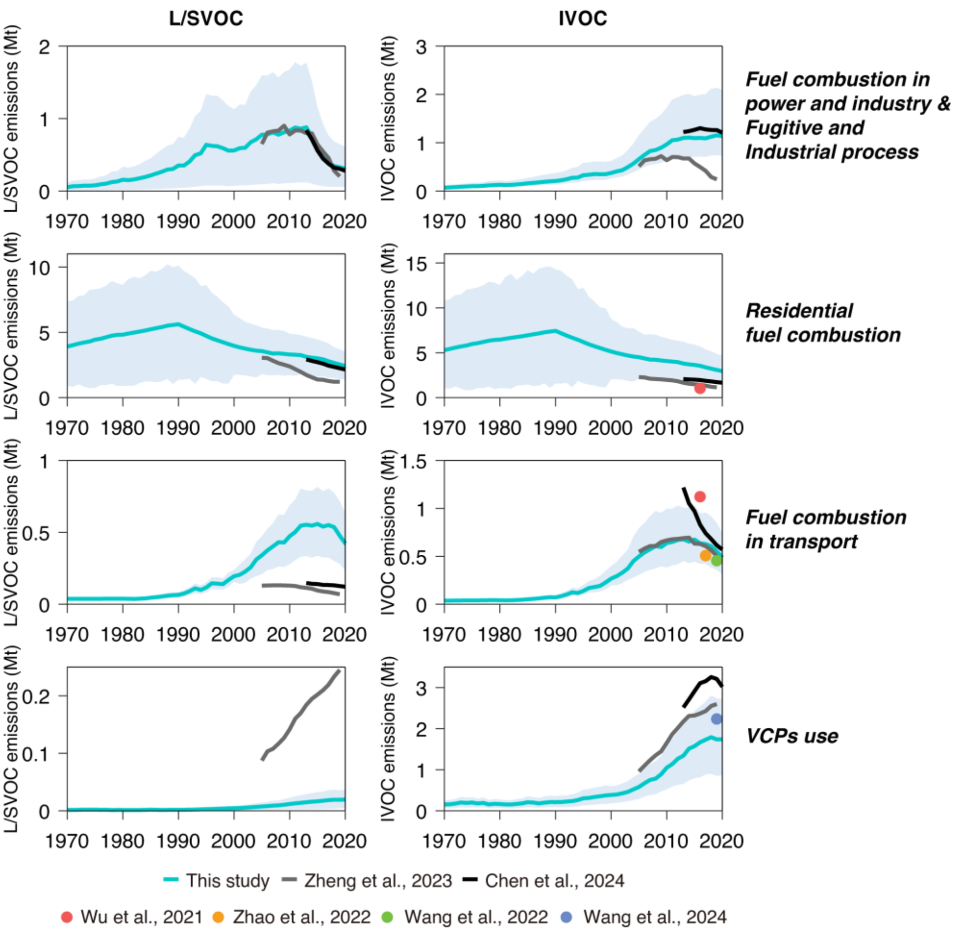

Figure S6. The same as Fig. 6e and 6f but for sectoral L/SVOC (left column) and IVOC emissions (right column) in China.

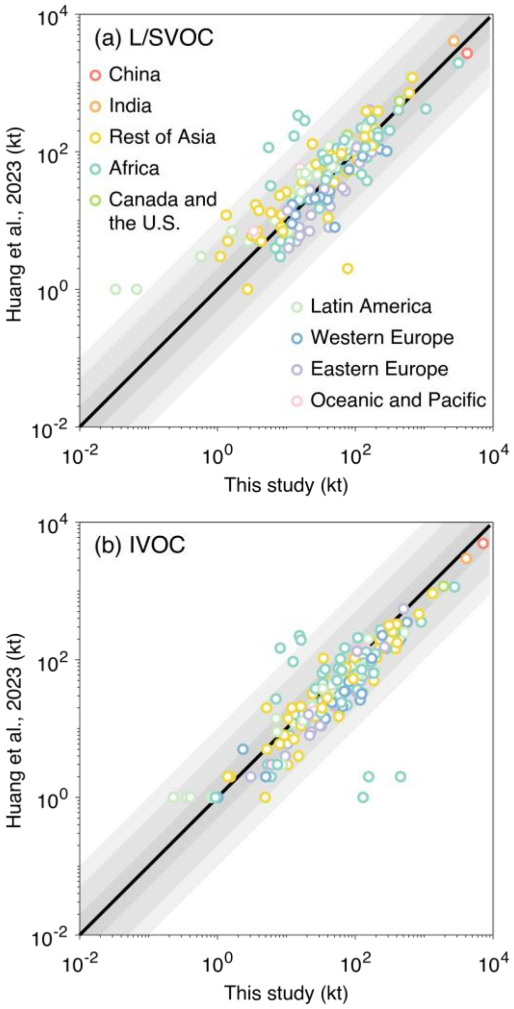

Figure S7. Comparison of country-level (a) L/SVOC and (b) IVOC emissions between this study and Huang et al. (2023) [33]. The solid black lines represent the 1:1 line, and the grey shaded areas represent 2:1 (1:2), 5:1 (1:5), and 10:1 (1:10) ranges, respectively. Scatter colors represent the regions to which the countries/territories belong.

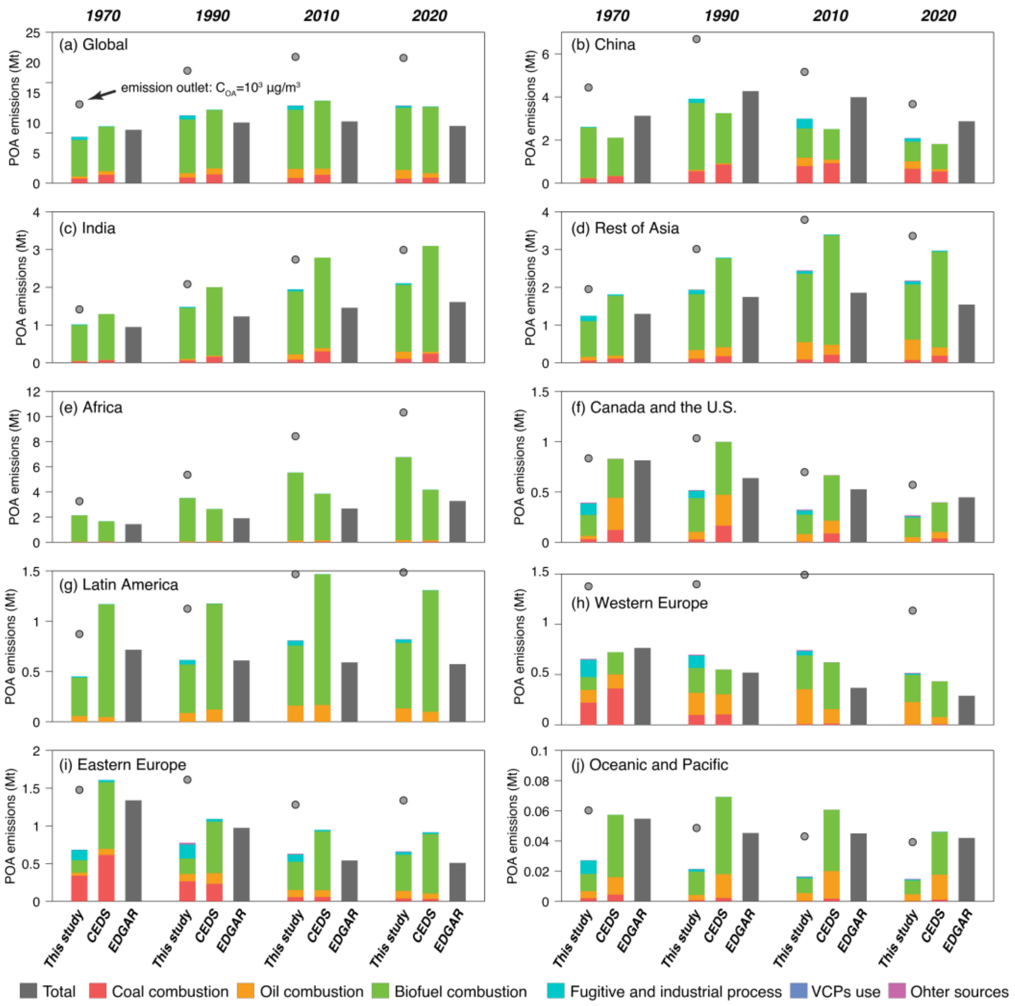

Figure S8. Comparison of global and regional particle-phase L/S/IVOC emissions in this study with POA emissions from the EDGAR and CEDS inventory. For this study, the bars represent particle-phase emissions in the ambient atmosphere, while the grey scatters indicate emissions at the emission outlets (assuming $C_{OA}=10^3$ μg/m^3). Particle-phase emissions are estimated using the approach in Donahue et al. (2006) [29], and regional OA concentrations (C_{OA}) are taken from Huang et al. (2023) [33]. OC emissions from EDGAR and CEDS are converted to POA using an OM-to-OC ratio of 1.4. EDGAR emissions are not accumulated by major sector due to incomplete information.

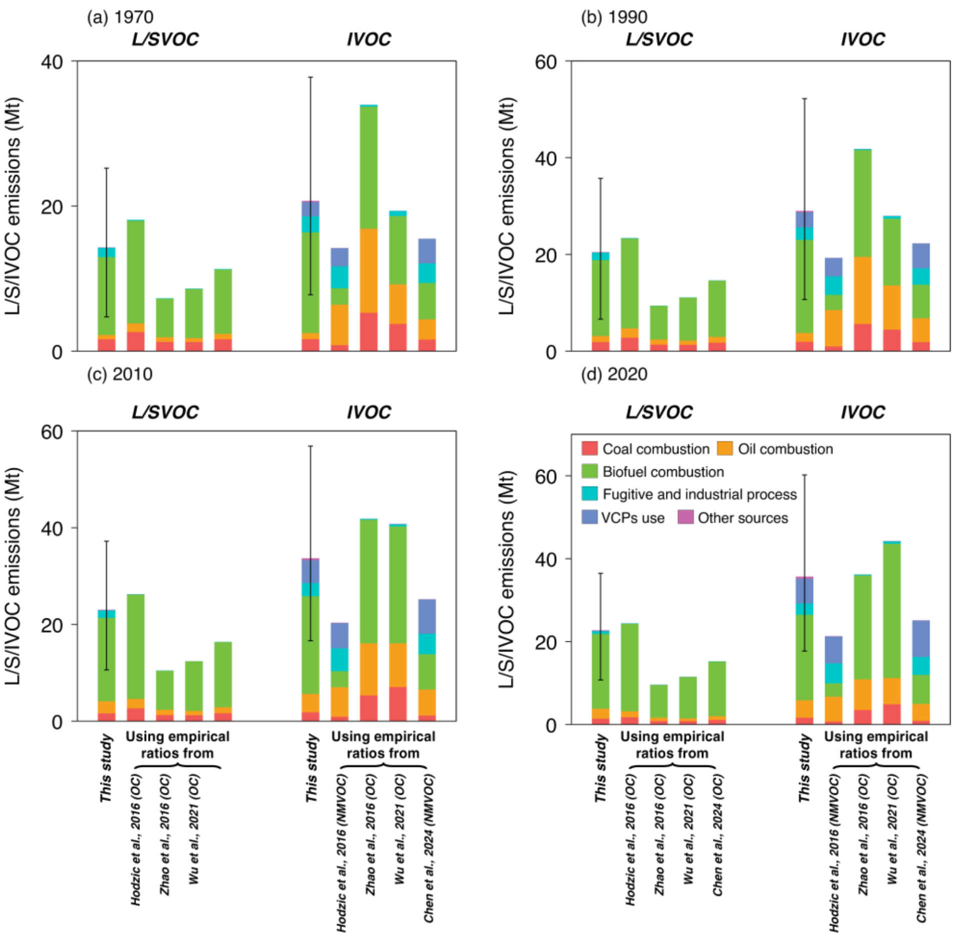

Figure S9. Comparison of global L/SVOC and IVOC emissions between this study and the estimates derived by using empirical-ratio-based approaches in (a) 1970, (b) 1990, (c) 2010, and (d) 2020. Error bars represent the uncertainty ranges of the MEIC-global emission estimates. The brackets following the references indicate the types of baseline emissions (OC and/or NMVOC).

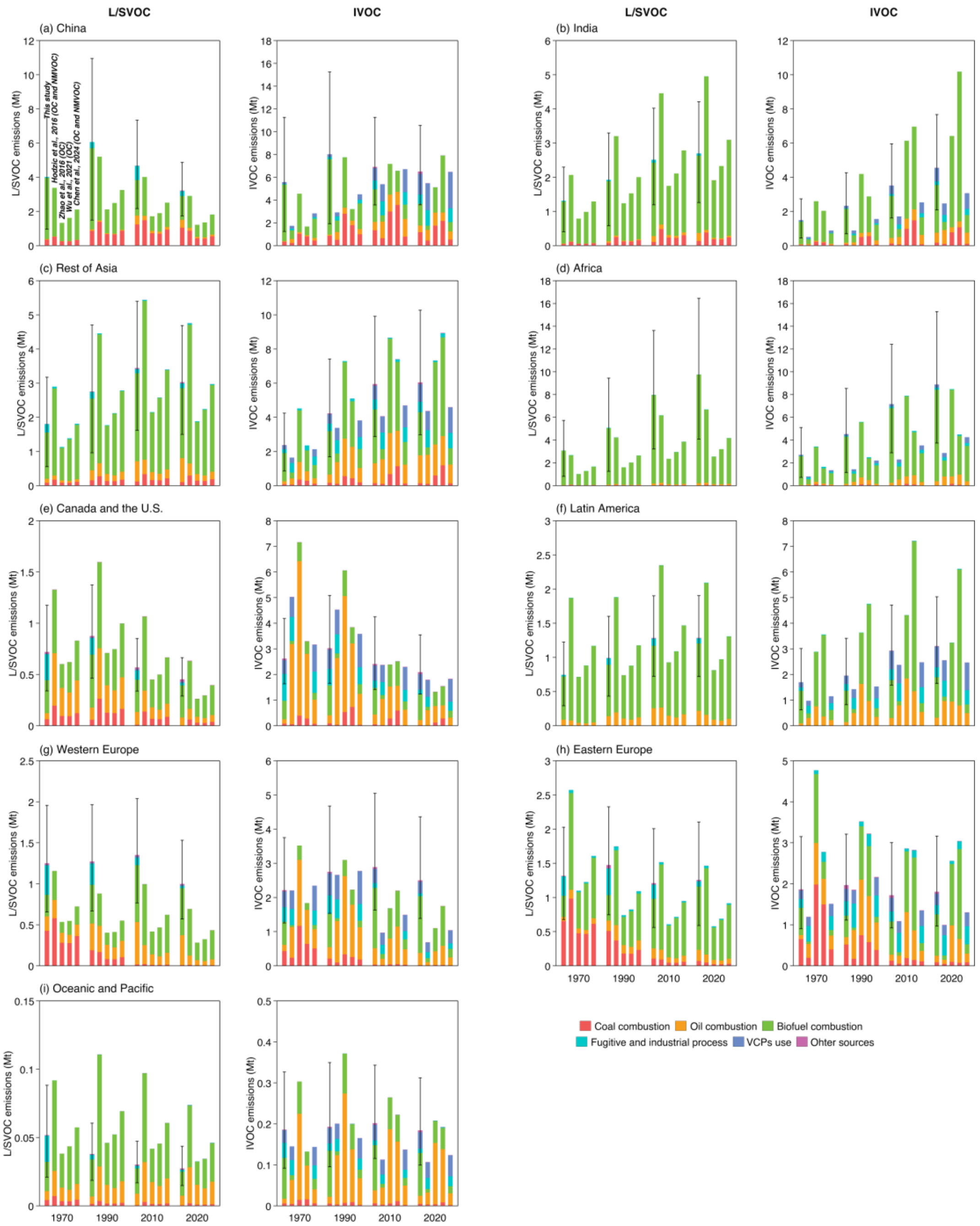

Figure S10. The same as Fig. S9 but at regional scale.

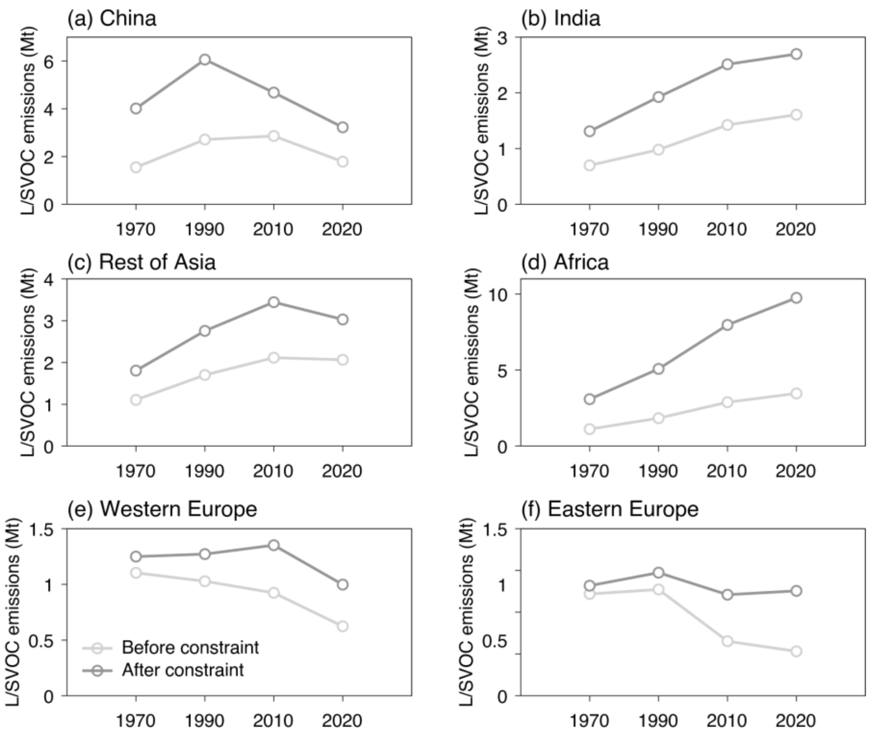

Figure S11. Comparison of regional emission estimates before and after constraining emission factors for residential biofuel combustion.

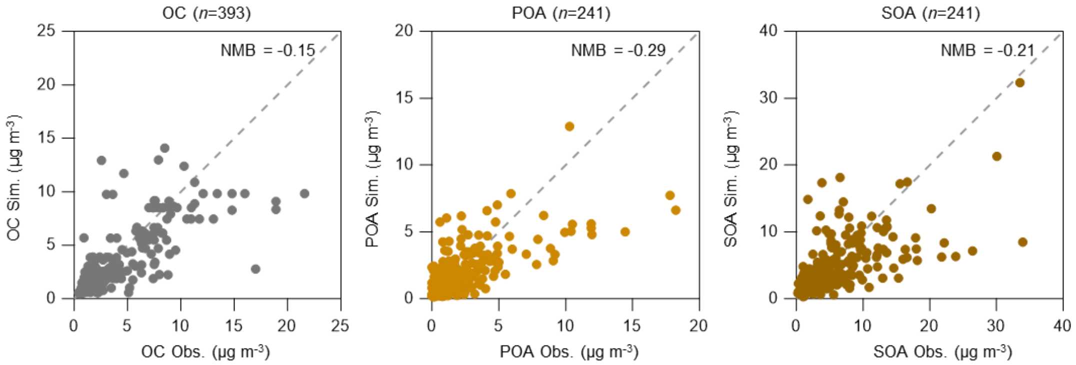

Figure S12. Comparison of observed and simulated OC, POA, and SOA concentrations.

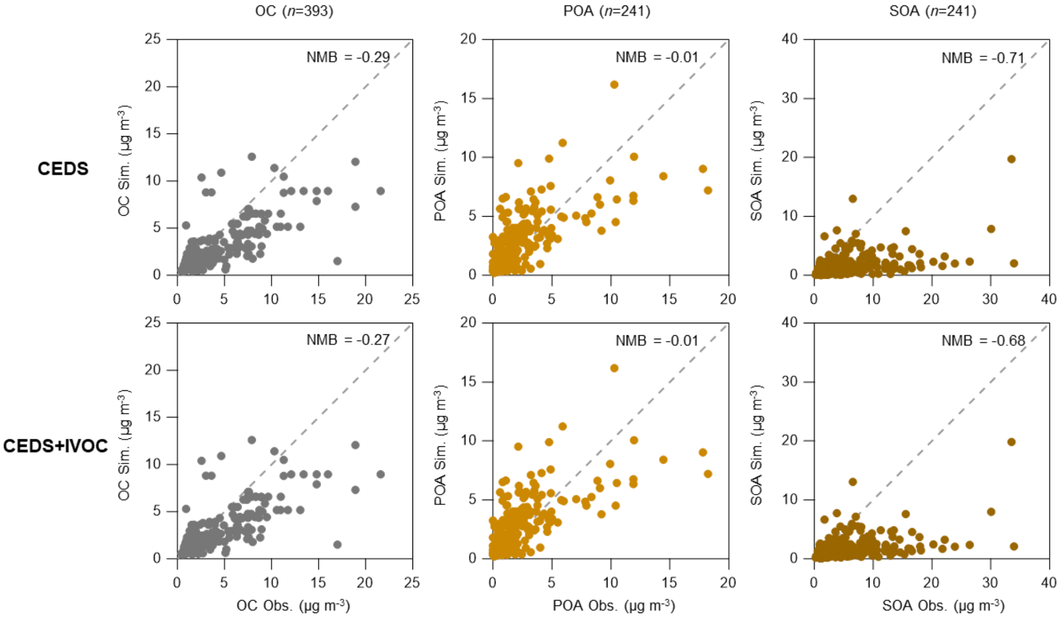

Figure S13. Comparison of observed and simulated OC, POA, and SOA concentrations for the CEDS and CEDS+IVOC simulations.

Table S1. The source categories and mapping to the major and detailed sources in the analysis.

<table>
<tr><th>1st level</th><th>2nd level</th><th>3rd level</th><th>4th level</th><th>5th level</th><th>Major sector</th><th>Detailed sources</th></tr>
<tr><td rowspan="24">Energy-power and industry</td><td>Power generation</td><td rowspan="10">Coal (Anthracite, Coking coal, Other bituminous coal, Sub-bituminous coal, Lignite, Patent fuel, Coke oven coke, Gas coke, Coal tar, BKB, Gas works gas, Coke oven gas, Blast furnace gas, Other recovered gases, Peat, Peat products)</td><td rowspan="24">None</td><td rowspan="24">None</td><td rowspan="24">Mapped to coal combustion, oil combustion, biofuel combustion, and other sources according to fuel types</td><td>Power</td></tr>
<tr><td>Heat (auto producer)</td><td rowspan="23">Industry</td></tr>
<tr><td>Heat (public)</td></tr>
<tr><td>Coal mines</td></tr>
<tr><td>Oil and gas extraction</td></tr>
<tr><td>Blast furnaces</td></tr>
<tr><td>Gas works</td></tr>
<tr><td>Gasification plants for biogases</td></tr>
<tr><td>Coke ovens</td></tr>
<tr><td>Patent fuel plants</td></tr>
<tr><td>BKB/peat briquette plants</td><td rowspan="14">Oil (Crude oil, Natural gas liquids, Refinery feedstocks, Additives/blending components, Other hydrocarbons, Refinery gas, Ethane, Liquefied petroleum gases (LPG), Motor gasoline excluding biofuels, Aviation gasoline, Gasoline type jet fuel, Kerosene type jet fuel excluding biofuels, Other kerosene, Gas/diesel oil excluding biofuels, Fuel oil, Naphtha, White spirit and SBP, Lubricants, Bitumen, Paraffin waxes, Petroleum coke, Other oil products, Oil shale</td></tr>
<tr><td>Oil refineries</td></tr>
<tr><td>Coal liquefaction plants</td></tr>
<tr><td>Liquefaction (LNG) / regasification plants</td></tr>
<tr><td>Gas-to-liquids (GTL) plants</td></tr>
<tr><td>Own use in electricity, CHP and heat plants</td></tr>
<tr><td>Charcoal production plants</td></tr>
<tr><td>Non-specified transformation industries</td></tr>
<tr><td>Iron and steel</td></tr>
<tr><td>Non-ferrous metals</td></tr>
<tr><td>Chemicals</td></tr>
<tr><td>Pulp and paper</td></tr>
<tr><td>Food and tobacco</td></tr>
<tr><td>Cement</td></tr>
</table>

	Other non-metallic minerals	and oil sands)			
	Transport equipment	Natural gas (Natural gas)			
	Machinery				
	Mining and quarrying	Biofuel (Renewable municipal waste, Primary solid biofuels, Biogases, Bio gasoline, Biodiesels, Bio jet kerosene, Other liquid biofuels, Non-specified primary biofuels/waste, Charcoal)			
	Wood products				
	Construction				
	Textile and leather				
	Other non-specified industries				
Energy-transport	International aviation		LTO and cruise		Mapped to off-road gasoline, off-road diesel, and other transport
	Domestic aviation				
	Rail		None		
	International navigation				
	Domestic navigation				
	Pipeline transport				
	Other non-specified transport				
	Agriculture and forestry		Emission standards	Implied in emission factors	
	Fishing				
	Cars		Running, start, evaporation (Stage 0, Stage 1, Stage 2, Stage 3, Stage 4, Stage 5, Stage 6, Stage 7)	Implied in emission factors	Mapped to on-road gasoline, on-road diesel, and other transport
	Light duty trucks				
	Buses				
	Heavy duty trucks				
	Motorcycles				
	Other fleet totals				
Energy-commercial and residential	Commercial and institutional		Traditional stoves and advanced stoves	None	Residential
	Residential (rural)				
	Residential (urban)				
	Non-specified sectors		None	None	

	Fugitive (solid fuel production)	Coke oven coke production	None	None		Fugitive - solid fuel production
Energy-fugitive	Fugitive (oil and gas)	Oil production	None	Abatement		Fugitive-oil and gas
		Oil storage and transport				
		Oil refining				
		Gasoline storage				
		Gasoline distribution				
		Diesel storage				
		Venting and flaring				
		Flaring in oil refinery				
		Natural gas production, processing, storage, and distribution				
		Gas venting and flaring				
Industrial process	Mineral industry	Cement production	None	None	Fugitive and industrial process	Other industries
		Clicker production				
		Glass production				
		Lime production				
		Brick production				
	Chemical industry (inorganic)	Ammonia	None	Abatement		Chemical industry
		Carbon black				
	Chemical industry (organic)	Adipic acid	None	Abatement		
		Ethylene				
		Vinyl chloride				
		Styrene				
		Low density polyethylene (LDPE)				
		High density polyethylene (HDPE)				
		Polyvinyl chloride (PVC)				
		Polypropylene (PP)				
		Polystyrene (PS)				
		SAN & ABS resins				
		Other synthetic resins				

		Ethylene oxide				
		Methanol				
		Acrylonitrile				
		Glyoxylic acid				
	Chemical industry (manufacturing)	Synthetic rubber	None	Abatement		
		Tyre production				
		Pharmaceutical production				
		Asphalt				
		Paint production				
		Printing ink production				
		Glues production				
		Shoes production				
		Leather tanning				
		Synthetic fibre				
		Wool				
		Silk				
		Cloth				
		Artificial fibre				
	Metal industry	Sinter production	None	None		Metal industry
		Pellet production				
		Pig iron production				
		DRI production				
		Steel production	EAF/BOF/OHF			
	Pulp, paper, and food industry	Paper pulp	None	Abatement		Other industries
		Paper				
		Plywood				
		Bread				
		Biscuit				
		Sugar				
		Flour				
		Oilseed				
		Beer				
		Wine				
		Spirit				
VCPs use	Paint use	Architectural interior wall	Waterborne and solvent-based paints	Abatement	VCPs use	Architectural paint
		Architecture other				
		Vehicle manufacturing				Other paint
		Vehicle repairing				
		Wood				

		Other industrial paint				
	Other industrial VCPs use	Vehicle dewax	None	Abatement		Other VCPs
		Vehicle reseal				
		Printing ink use				
		Glues and adhesives use				
		Preservation of wood				
		Degreasing				
		Dry cleaning				
	Domestic use	Domestic VCPs use	None	Abatement		Domestic VCPs
		Pesticide				Other VCPs

Table S2. The countries/territories, their ISO-3166 codes, and mapping to the regions in the analysis.

Country/territory	ISO-3166 code	Region
Afghanistan	AFG	Rest of Asia
Albania	ALB	Eastern Europe
Algeria	DZA	Africa
American Samoa	ASM	Oceania and Pacific
Angola	AGO	Africa
Anguilla	AIA	Latin America
Antigua and Barbuda	ATG	Latin America
Argentina	ARG	Latin America
Armenia	ARM	Rest of Asia
Aruba	ABW	Latin America
Australia	AUS	Oceania and Pacific
Austria	AUT	Western Europe
Azerbaijan	AZE	Rest of Asia
Bahamas	BHS	Latin America
Bahrain	BHR	Rest of Asia
Bangladesh	BGD	Rest of Asia
Barbados	BRB	Latin America
Belarus	BLR	Eastern Europe
Belgium	BEL	Western Europe
Belize	BLZ	Latin America
Benin	BEN	Africa
Bermuda	BMU	Latin America
Bhutan	BTN	Rest of Asia
Bolivia	BOL	Latin America
Bonaire, Sint Eustatius and Saba	BES	Latin America
Bosnia and Herzegovina	BIH	Eastern Europe
Botswana	BWA	Africa
Brazil	BRA	Latin America
Brunei Darussalam	BRN	Rest of Asia
Bulgaria	BGR	Eastern Europe
Burkina Faso	BFA	Africa
Burundi	BDI	Africa
Cape Verde	CPV	Africa
Cambodia	KHM	Rest of Asia
Cameroon	CMR	Africa
Canada	CAN	Canada and the U.S.
Cayman Islands	CYM	Latin America
Central African Republic	CAF	Africa
Chad	TCD	Africa
Chile	CHL	Latin America

China (including Hong Kong, Macao, and Taiwan)	CHN	China
Colombia	COL	Latin America
Comoros	COM	Africa
Democratic Republic of Congo	COD	Africa
Congo	COG	Africa
Cook Islands	COK	Oceania and Pacific
Costa Rica	CRI	Latin America
Croatia	HRV	Eastern Europe
Cuba	CUB	Latin America
Curacao	CUW	Latin America
Cyprus	CYP	Eastern Europe
Czech Republic	CZE	Eastern Europe
Cote d'Ivoire	CIV	Africa
Denmark	DNK	Western Europe
Djibouti	DJI	Africa
Dominica	DMA	Latin America
Dominican Republic	DOM	Latin America
Ecuador	ECU	Latin America
Egypt	EGY	Africa
El Salvador	SLV	Latin America
Equatorial Guinea	GNQ	Africa
Eritrea	ERI	Africa
Estonia	EST	Eastern Europe
Swaziland	SWZ	Africa
Ethiopia	ETH	Africa
Falkland Islands	FLK	Latin America
Faeroe Islands	FRO	Western Europe
Fiji	FJI	Oceania and Pacific
Finland	FIN	Western Europe
France	FRA	Western Europe
French Guiana	GUF	Latin America
French Polynesia	PYF	Oceania and Pacific
Gabon	GAB	Africa
Gambia	GMB	Africa
Georgia	GEO	Rest of Asia
Germany	DEU	Western Europe
Ghana	GHA	Africa
Gibraltar	GIB	Western Europe
Greece	GRC	Western Europe
Greenland	GRL	Western Europe
Grenada	GRD	Latin America
Guadeloupe	GLP	Latin America
Guam	GUM	Oceania and Pacific

Guatemala	GTM	Latin America
Guinea	GIN	Africa
Guinea-Bissau	GNB	Africa
Guyana	GUY	Latin America
Haiti	HTI	Latin America
Honduras	HND	Latin America
Hungary	HUN	China
Iceland	ISL	Eastern Europe
India	IND	Western Europe
Indonesia	IDN	India
Islamic Republic of Iran	IRN	Rest of Asia
Iraq	IRQ	Rest of Asia
Ireland	IRL	Rest of Asia
Isle of Man	IMN	Western Europe
Israel	ISR	Western Europe
Italy	ITA	Rest of Asia
Jamaica	JAM	Western Europe
Japan	JPN	Latin America
Jersey	JEY	Rest of Asia
Jordan	JOR	Western Europe
Kazakhstan	KAZ	Rest of Asia
Kenya	KEN	Rest of Asia
Kiribati	KIR	Africa
Democratic People’s Republic of Korea	PRK	Oceania and Pacific
Republic of Korea	KOR	Rest of Asia
Kuwait	KWT	Rest of Asia
Kyrgyzstan	KGZ	Rest of Asia
Laos	LAO	Rest of Asia
Latvia	LVA	Rest of Asia
Lebanon	LBN	Eastern Europe
Lesotho	LSO	Rest of Asia
Liberia	LBR	Africa
Libya	LBY	Africa
Liechtenstein	LIE	Africa
Lithuania	LTU	Western Europe
Luxembourg	LUX	Eastern Europe
Madagascar	MDG	Western Europe
Malawi	MWI	China
Malaysia	MYS	Africa
Maldives	MDV	Africa
Mali	MLI	Rest of Asia
Malta	MLT	Rest of Asia
Marshall Islands	MHL	Africa

Martinique	MTQ	Western Europe
Mauritania	MRT	Oceania and Pacific
Mauritius	MUS	Latin America
Mayotte	MYT	Africa
Mexico	MEX	Africa
Federated States of Micronesia	FSM	Africa
Moldova	MDA	Latin America
Mongolia	MNG	Oceania and Pacific
Montenegro	MNE	Eastern Europe
Montserrat	MSR	Rest of Asia
Morocco	MAR	Eastern Europe
Mozambique	MOZ	Latin America
Myanmar	MMR	Africa
Namibia	NAM	Africa
Nauru	NRU	Rest of Asia
Nepal	NPL	Africa
Netherlands	NLD	Oceania and Pacific
New Caledonia	NCL	Rest of Asia
New Zealand	NZL	Western Europe
Nicaragua	NIC	Oceania and Pacific
Niger	NER	Oceania and Pacific
Nigeria	NGA	Latin America
Niue	NIU	Africa
Norfolk Island	NFK	Africa
North Macedonia	MKD	Oceania and Pacific
Northern Mariana Islands	MNP	Oceania and Pacific
Norway	NOR	Eastern Europe
Oman	OMN	Oceania and Pacific
Pakistan	PAK	Western Europe
Palau	PLW	Rest of Asia
Palestine	PSE	Rest of Asia
Panama	PAN	Oceania and Pacific
Papua New Guinea	PNG	Rest of Asia
Paraguay	PRY	Latin America
Peru	PER	Oceania and Pacific
Philippines	PHL	Latin America
Pitcairn	PCN	Latin America
Poland	POL	Rest of Asia
Portugal	PRT	Oceania and Pacific
Puerto Rico	PRI	Eastern Europe
Qatar	QAT	Western Europe
Romania	ROU	Latin America
Russia	RUS	Rest of Asia

Rwanda	RWA	Eastern Europe
Reunion	REU	Eastern Europe
Saint Helena	SHN	Africa
Saint Kitts and Nevis	KNA	Africa
Saint Lucia	LCA	Africa
Saint Pierre and Miquelon	SPM	Latin America
Saint Vincent and Grenadines	VCT	Latin America
Samoa	WSM	Latin America
Sao Tome and Principe	STP	Latin America
Saudi Arabia	SAU	Oceania and Pacific
Senegal	SEN	Africa
Serbia	SRB	Rest of Asia
Seychelles	SYC	Africa
Sierra Leone	SLE	Eastern Europe
Singapore	SGP	Africa
Sint Maarten	SXM	Africa
Slovakia	SVK	Rest of Asia
Slovenia	SVN	Latin America
Solomon Islands	SLB	Eastern Europe
Somalia	SOM	Eastern Europe
South Africa	ZAF	Oceania and Pacific
South Sudan	SSD	Africa
Spain	ESP	Africa
Sri Lanka	LKA	Africa
Sudan	SDN	Western Europe
Suriname	SUR	Rest of Asia
Sweden	SWE	Africa
Switzerland	CHE	Latin America
Syria	SYR	Western Europe
Tajikistan	TJK	Western Europe
Tanzania	TZA	Rest of Asia
Thailand	THA	China
Timor-Leste	TLS	Rest of Asia
Togo	TGO	Africa
Tokelau	TKL	Rest of Asia
Tonga	TON	Rest of Asia
Trinidad and Tobago	TTO	Africa
Tunisia	TUN	Oceania and Pacific
Turkey	TUR	Oceania and Pacific
Turkmenistan	TKM	Latin America
Turks and Caicos Islands	TCA	Africa
Tuvalu	TUV	Rest of Asia
Uganda	UGA	Rest of Asia

Ukraine	UKR	Latin America
United Arab Emirates	ARE	Oceania and Pacific
United Kingdom	GBR	Africa
United States	USA	Eastern Europe
Uruguay	URY	Rest of Asia
Uzbekistan	UZB	Western Europe
Vanuatu	VUT	Canada and the U.S.
Venezuela	VEN	Latin America
Vietnam	VNM	Rest of Asia
British Virgin Islands	VGB	Oceania and Pacific
United States Virgin Islands	VIR	Latin America
Wallis and Futuna Islands	WLF	Rest of Asia
Western Sahara	ESH	Latin America
Yemen	YEM	Latin America
Zambia	ZMB	Oceania and Pacific
Zimbabwe	ZWE	Africa

Table S3. Definition of volatility bins in this study.

	Volatility bin	C^* range
LVOC	≤-2	$C^* \leq 0.03$ μg/m^3
	-1	0.03 μg/m^3 < $C^* \leq 0.3$ μg/m^3
SVOC	0	0.3 μg/m^3 < $C^* \leq 3$ μg/m^3
	1	3 μg/m^3 < $C^* \leq 30$ μg/m^3
	2	30 μg/m^3 < $C^* \leq 300$ μg/m^3
IVOC	3	300 μg/m^3 < $C^* \leq 3000$ μg/m^3
	4	3000 μg/m^3 < $C^* \leq 3\times10^4$ μg/m^3
	5	3×10^4 μg/m^3 < $C^* \leq 3\times10^5$ μg/m^3
	6	3×10^5 μg/m^3 < $C^* \leq 3\times10^6$ μg/m^3
NMVOC	7	$C^* > 3\times10^6$ μg/m^3

Table S4. Unabated emission factors.

						$\log_{10}C^*$								
**Major sector**	**Source**	**Region[a]**	**Refs**	**Confidence level[b]**	**Unit**	**≤-2**	**-1**	**0**	**1**	**2**	**3**	**4**	**5**	**6**
Coal combustion	Power /Industry	-	*6*	D	g/kg	0	0	0	0.0009	0.0019	0.0056	0.008	0.0183	0.0122
	Residential-bituminous coal	-	*6,7,8*	A+ for China and B for other regions	g/kg	0.77	0.44	0.35	1.09	1.06	1.21	0.86	0.76	0.72
	Residential-anthracite	-	*6,7,8*		g/kg	0.0057	0.0004	0.003	0.0024	0.0024	0.0082	0.0152	0.0105	0.0062
	Residential-other coal	-	*6,7,8*		g/kg	0.77	0.44	0.35	1.09	1.06	1.21	0.86	0.76	0.72
Oil combustion	Power /Industry	-	*6*	D	g/kg	0.0077	0.0044	0.0013	0.0027	0.0166	0.0189	0.0186	0.0254	0.0154
	Residential	-	*6*	D	g/kg	0.0101	0.0008	0.0054	0.0042	0.0044	0.017	0.0454	0.0738	0.05
	Transport-aviation (LTO)	-	*20*	C	g/kg	0.007	0.001	0.004	0.007	0.012	0.063	0.101	0.015	0.023
	Transport-aviation (cruise)	-	*20*	C	g/kg	0.0074	0.0006	0.0040	0.0073	0.0020	0.0001	0.0003	0.0110	0.0295
	Transport-navigation	-	*19*	A+ for China and B for other regions	g/kg	1.05	0.15	0.14	0.16	0.18	0.23	0.36	0.25	0.17
	Transport-car-gasoline[c]	developing	*13*	A+ for China and B for other regions	g/kg	0.0021-0.0045	0.0004-0.0009	0.0014-0.0031	0.0079-0.0168	0.0055-0.0116	0.0013-0.0049	0.0092-0.0301	0.0153-0.0493	0.0271-0.0685
		developed	*12*	A+ for the U.S. and B for other regions	g/kg	0.001-0.0065	0.0002-0.0012	0.0007-0.0045	0.0038-0.0244	0.0026-0.0169	0.0026-0.0066	0.0023-0.0111	0.0048-0.0339	0.0191-0.1342
	Transport-car-diesel[c]	developing	*14,15*	A+ for China and B for other regions	g/kg	0.05-0.63	0.07-0.34	0.32-0.5	0.36-0.61	0.31-0.55	0.02-0.06	0.1-0.26	0.25-0.34	0.61-1.01
		developed	*11*	A+ for the U.S. and B for other regions	g/kg	0-0.0527	0.0001-0.0109	0.0002-0.0124	0.0115-0.0388	0.0058-0.0388	0.0035-0.1041	0.0081-0.3169	0.0148-0.502	0.0228-0.4739
	Transport-bus-gasoline[c]	developing	*13*	A+ for China and B for other regions	g/kg	0.0025-0.0053	0.0005-0.001	0.0017-0.0037	0.0095-0.0201	0.0066-0.014	0.0016-0.0059	0.0111-0.0361	0.0183-0.0592	0.0325-0.0822
		developed	*12*	A+ for the U.S. and B for other regions	g/kg	0.0012-0.0078	0.0002-0.0015	0.0008-0.0054	0.0045-0.0292	0.0031-0.0203	0.0031-0.0079	0.0028-0.0133	0.0057-0.0406	0.0229-0.161
	Transport-bus-diesel[c]	developing	*14,15*	A+ for China and B for other regions	g/kg	0.01-1.22	0.02-0.48	0.13-0.75	0.16-0.85	0.11-0.77	0.07-0.3	0.1-0.79	0.08-0.68	0.08-1.17
		developed	*11*	A+ for the U.S. and B	g/kg	0-0.0611	0.0001	0.0002	0.0133	0.0068	0.0041	0.0094	0.0172	0.0264

				for other regions			-0.0126	-0.0144	-0.045	-0.045	-0.1208	-0.3676	-0.5823	-0.5497
	Transport-LDT-gasoline[c]	developing	*13*	A+ for China and B for other regions	g/kg	0.0021-0.0045	0.0004-0.0009	0.0014-0.0031	0.0079-0.0168	0.0055-0.0116	0.0013-0.0049	0.0092-0.0301	0.0153-0.0493	0.0271-0.0685
		developed	*12*	A+ for the U.S. and B for other regions	g/kg	0.001-0.0114	0.0002-0.0022	0.0007-0.0079	0.0036-0.0429	0.0025-0.0297	0.0035-0.0143	0.0029-0.021	0.0066-0.0651	0.0143-0.2263
	Transport-LDT-diesel[c]	developing	*14,15*	A+ for China and B for other regions	g/kg	0.05-0.63	0.07-0.34	0.32-0.5	0.36-0.61	0.31-0.55	0.02-0.06	0.1-0.26	0.25-0.34	0.61-1.01
		developed	*11*	A+ for the U.S. and B for other regions	g/kg	0-0.0527	0.0001-0.0109	0.0002-0.0124	0.0115-0.0388	0.0058-0.0388	0.0035-0.1041	0.0081-0.3169	0.0148-0.502	0.0228-0.4739
	Transport-HDT-gasoline[c]	developing	*13*	A+ for China and B for other regions	g/kg	0.0482-0.0586	0.0093-0.0113	0.0334-0.0406	0.1818-0.221	0.1261-0.1533	0.0213-0.0765	0.1228-0.3126	0.3006-0.4066	0.7294-1.2103
		developed	*12*	A+ for the U.S. and B for other regions	g/kg	0.0011-0.0136	0.0002-0.0026	0.0008-0.0094	0.0043-0.0514	0.003-0.0357	0.0042-0.0172	0.0035-0.0252	0.008-0.0781	0.0171-0.2715
	Transport-HDT-diesel[c]	developing	*14,15*	A+ for China and B for other regions	g/kg	0.01-1.22	0.02-0.48	0.13-0.75	0.16-0.85	0.11-0.77	0.07-0.3	0.1-0.79	0.08-0.68	0.08-1.17
		developed	*11*	A+ for the U.S. and B for other regions	g/kg	0-0.0611	0.0001-0.0126	0.0002-0.0144	0.0133-0.045	0.0068-0.045	0.0041-0.1208	0.0094-0.3676	0.0172-0.5823	0.0264-0.5497
	Transport-motorcycle	-	*6,33*	D	g/kg	0.253	0.3542	0.4554	0.8096	1.3156	1.815	2.343	4.9544	19.16
	Transport-LPG vehicles	-	*6,33*	D	g/kg	0.00065	0.00065	0.0026	0.01385	0.0119	0.10155	0.2323	0.36935	0.3591
	Transport-off road machinery-diesel (agriculture)	developing	*18*	A+ for China and B for other regions	g/kg	0.0017	0.0207	0.0075	0.0116	0.0141	0.0108	0.0274	0.0804	0.2682
		developed	*11*	A+ for the U.S. and B for other regions	g/kg	0.0261	0.0054	0.0061	0.0192	0.0192	0.0360	0.1345	0.2455	0.2750
	Transport-off road machinery-diesel (other)	developing	*17*	A+ for China and B for other regions	g/kg	0.0000	0.0133	0.0248	0.0520	0.1454	0.1194	0.2923	0.3607	0.3935
		developed	*11*	A+ for the U.S. and B for other regions	g/kg	0.0261	0.0054	0.0061	0.0192	0.0192	0.0360	0.1345	0.2455	0.2750
	Transport-off road machinery-gasoline	-	*12*	A+ for the U.S. and B for other regions	g/kg	0.1091	0.0210	0.0755	0.4111	0.2853	0.0715	0.1804	0.5528	2.3289
Biofuel combustion	Residential	South Asia	*24,25*	A+ for India and A for other countries	g/kg	1.81	0.79	0.93	1.18	2.23	1.15	1.72	1.83	3.31
		China	*22,23*	A+	g/kg	Constrained by OC emission factors for crop and wood combustion from MEIC-China and varied year by								

									year					
		Africa	22,23,24,25	A	g/kg	3.56	1.57	1.27	1.63	3.98	1.60	2.37	2.34	3.79
		Europe	21	A	g/kg	Constrained by BBOA ratios in Table S9								
		Other developing	22,23,24,25	B	g/kg	1.18	0.52	0.42	0.54	1.32	1.60	2.37	2.34	3.79
		Other developed	21	A+ for the U.S. and B for other countries	g/kg	0.03	0.33	0.42	0.71	0.66	1.62	0.70	1.59	12.09
	Sinter (end of pipe)	-	1,2,48	C	g/kg	0.0110	0.0063	0.0019	0.0028	0.0219	0.0071	0.0026	0.0315	0.0114
	Sinter (fugitive)	-	1,48	C	g/kg	0.0021	0.0012	0.0004	0.0005	0.0041	0	0	0	0
	Pellet (end of pipe)	-	1,48	C	g/kg	0.0110	0.0063	0.0019	0.0028	0.0219	0.0007	0.0003	0.0032	0.0012
	Pellet (fugitive)	-	1,48	C	g/kg	0.0021	0.0012	0.0004	0.0005	0.0041	0	0	0	0
	Steel (BOF)	-	1,48	C	g/kg	0.8866	0.5051	0.1554	0.2261	1.7590	0.0014	0.0005	0.0062	0.0022
	Steel (EAF)	-	1,48	C	g/kg	0.0764	0.0435	0.0134	0.0195	0.1515	0.0024	0.0009	0.0105	0.0038
	Steel (OHF)	-	1,48	C	g/kg	0.5370	0.3060	0.0941	0.1369	1.0655	0.0014	0.0005	0.0062	0.0022
	Cement	-	10,44	C	g/kg	0.0093	0.0053	0.0016	0.0024	0.0185	0	0	0	0
	Lime	-	42,43,49	C	g/kg	0.0059	0.0034	0.0010	0.0015	0.0118	0	0	0	0
	Brick	Country-specific	42,43,49	C	g/kg	Vary by country according to brick kiln types								
Fugitive and industrial process	Coke	-	1,48	C	g/kg	0.2291	0.1305	0.0402	0.0584	0.4545	0.0696	0.0258	0.3070	0.1110
	Oil refining	-	2	C	g/kg	0	0	0	0	0	0.0051	0.0025	0.0301	0.0648
	Ammonia	-	2	C	g/kg	0	0	0	0	0	0.0015	0.0001	0.0340	0.3198
	Carbon black	-	2	C	g/kg	0	0	0	0	0	0.0169	0.0015	0.3862	3.6298
	Adipic acid	-	2	C	g/kg	0	0	0	0	0	0.0015	0.0001	0.0345	0.3244
	Ethylene	-	2	C	g/kg	0	0	0	0	0	0.0002	0.0000	0.0043	0.0407
	Vinyl Chloride	-	2	C	g/kg	0	0	0	0	0	0.0008	0.0001	0.0180	0.1694
	Styrene	-	2	C	g/kg	0	0	0	0	0	0.0003	0.0000	0.0072	0.0678
	Low density polyethylene	-	2	C	g/kg	0	0	0	0	0	0.0008	0.0001	0.0173	0.1626
	High density polyethylene	-	2	C	g/kg	0	0	0	0	0	0.0007	0.0001	0.0166	0.1558
	Polyvinyl chloride	-	2	C	g/kg	0	0	0	0	0	0.0027	0.0002	0.0613	0.5759
	Polypropylene	-	2	C	g/kg	0	0	0	0	0	0.0013	0.0001	0.0288	0.2710
	Polystyrene	-	2	C	g/kg	0	0	0	0	0	0.0067	0.0006	0.1525	1.4331
	SAN & ABS	-	2	C	g/kg	0	0	0	0	0	0.0009	0.0001	0.0216	0.2033

	resins													
	Other synthetic resins	-	*2*	C	g/kg	0	0	0	0	0	0.0025	0.0002	0.0577	0.5421
	Ethylene oxide	-	*2*	C	g/kg	0	0	0	0	0	0.0006	0.0001	0.0144	0.1355
	Methanol	-	*2*	C	g/kg	0	0	0	0	0	0.0005	0.0000	0.0108	0.1016
	Acrylonitrile	-	*2*	C	g/kg	0	0	0	0	0	0.0003	0.0000	0.0072	0.0678
	Glyoxylic acid	-	*2*	C	g/kg	0	0	0	0	0	0.0025	0.0002	0.0577	0.5421
	Synthetic rubber	-	*2*	C	g/kg	0	0	0	0	0	0.0025	0.0002	0.0577	0.5421
	Pharmaceutical Production	-	*2*	C	g/kg	0	0	0	0	0	0.0945	0.0083	2.1629	20.3274
	Asphalt	-	*2*	C	g/kg	0	0	0	0	0	0.0008	0.0001	0.0181	0.1705
	Paints production	-	*2*	C	g/g	0	0	0	0	0	0.0000	0.0000	0.0001	0.0010
	Printing ink production	-	*2*	C	g/kg	0	0	0	0	0	0.0189	0.0017	0.4326	4.0655
	Glues production	-	*2*	C	g/kg	0	0	0	0	0	0.0035	0.0003	0.0793	0.7453
	Shoes production	-	*2*	C	t/pair	0	0	0	0	0	0.0000	0.0000	0.0000	0.0000
	Leather tanning	-	*2*	C	g/g	0	0	0	0	0	0.0000	0.0000	0.0000	0.0000
	Synthetic fibres	-	*2*	C	g/kg	0	0	0	0	0	0.0031	0.0003	0.0721	0.6776
	Wool	-	*2*	C	g/kg	0	0	0	0	0	0.0031	0.0003	0.0721	0.6776
	Silk	-	*2*	C	g/kg	0	0	0	0	0	0.0031	0.0003	0.0721	0.6776
	Cloth	-	*2*	C	g/kg	0	0	0	0	0	0.0072	0.0006	0.1658	1.5584
	Artificial fibres	-	*2*	C	g/kg	0	0	0	0	0	0.0327	0.0029	0.7477	7.0265
	Paper pulp	-	*2*	C	g/kg	0	0	0	0	0	0.0000	0.0000	0.0000	0.0002
	Paper	-	*2*	C	g/kg	0	0	0	0	0	0.0000	0.0000	0.0000	0.0000
	Gasoline storage	-	*2,34*	C	g/kg	0	0	0	0	0	0.0530	0.0265	0.3126	0.6740
	Gasoline distribution	-	*2,34*	C	g/kg	0	0	0	0	0	0.1146	0.0572	0.6760	1.4573
	Diesel storage	-	*2,34*	C	g/kg	0	0	0	0	0	0.0001	0.0000	0.0003	0.0007
VCPs use	Architectural paint (water-borne)	-	*37,39*	B	g/kg	0	0	0	0	0	0	3.13	1.54	77.64
	Architectural	-	*37,39*	B	g/kg	0	0	0	0	0	0	3.17	5.77	274.60

	paint (solvent-borne)													
	Other paint (water-borne)	-	*37,40*	A+ for China and B for other regions	g/kg	0	0	0	0	0	0	4.90	8.48	4.08
	Other paint (solvent-borne)	-	*37,40*	A+ for China and B for other regions	g/kg	0	0	0	0	0	0	12.26	21.20	10.21
	Vehicle treated-dewax	-	*6,38*	D	kg/vehicle	0	0	0	0	0.0033	0.0165	0.0429	0.0528	0.2145
	Printing ink	-	*38,40*	B	g/kg	0	0	0	0	0	0	0	34.3	0
	Glues and adhesives	-	*38,40*	B	g/kg	0	0	0	0	2.31	4.03	2.91	19.97	8.41
	Degreasing	-	*38,40*	B	g/kg	0	0	0	0	0	0	0	27.30	68.25
	Dry cleaning	Country-specific	*38,40*	B	kg/capita	Vary by country and scale with NMVOC emission factors								
	Domestic Use	Country-specific	*38,40*	B	kg/capita	Vary by country and scale with NMVOC emission factors								
	Pesticides	-	*38,40*	B	g/kg	0	0	0	0	1.72	3.81	11.89	22.37	44.21
Other sources	Gas fuels[d]	-	*6,33*	D	g/m^3	0.0081-0.0082	0.0006-0.0047	0.0014-0.0043	0.0029-0.0034	0.0035-0.0179	0.0119-0.204	0.0202-0.0203	0.0199-0.0276	0.0122-0.0167
	Municipal waste	-	*6,33*	D	g/kg	0.0034	0.0036	0.0022	0.0103	0.0072	0.0105	0.0047	0.0046	0.0081

[a] When regions were not specified, global uniform values were used.

[b] A+: EFs measured locally; A: EFs measured in other regions but constrained by locally measured POA EFs or field observations; B: EFs measured in other regions without any constraints, or EFs derived by mass-balance-based estimates; C: EFs calculated from OC and NMVOC EFs; D: EFs lacking available data and taken from estimated values in literatures.

[c] Emission factors for on-road vehicles were emission-standard-specific.

[d] Gas fuels include natural gas, coke oven gas, blast furnace gas, and other recovered gases.

Table S5. The probability distributions of emission parameters.

1st level	2nd level	Activity rate	Technology distribution	Emission factor	Penetration ratios of control technology
Power generation	Coal (Anthracite, Coking coal, Other bituminous coal, Sub-bituminous coal, Lignite, Patent fuel, Coke oven coke, Gas coke, Coal tar, BKB, Gas works gas, Coke oven gas, Blast furnace gas, Other recovered gases, Peat, Peat products)	Normal Fossil fuel: 1970-1999 OECD countries CV=15%; non-OECD countries with local data fusion CV=20%; other countries CV=25% 2000-2020 OECD countries CV=10%; non-OECD countries with local data fusion CV=15%; other countries CV=20%'; Biomass: 1970-1999 OECD countries CV=40%; non-OECD countries with local data fusion CV=45%; other countries CV=50% 2000-2020 OECD countries CV=30%; non-OECD countries with local data fusion CV=35%; other countries CV=40%	None	Lognormal CV=12.5%	Uniform ±30%
Heat (auto producer)					
Heat (public)					
Coal mines					
Oil and gas extraction					
Blast furnaces					
Gas works					
Gasification plants for biogases					
Coke ovens					
Patent fuel plants					
BKB/peat briquette plants					
Oil refineries					
Coal liquefaction plants					
Liquefaction (LNG) / regasification plants	Oil (Crude oil, Natural gas liquids, Refinery feedstocks, Additives/blending components, Other hydrocarbons, Refinery gas, Ethane, Liquefied petroleum gases (LPG), Motor gasoline excluding biofuels, Aviation gasoline, Gasoline type jet fuel, Kerosene type jet fuel excluding biofuels, Other kerosene, Gas/diesel oil excluding biofuels, Fuel oil, Naphtha, White spirit and SBP, Lubricants, Bitumen,				
Gas-to-liquids (GTL) plants					
Own use in electricity, CHP and heat plants					
Charcoal production plants					
Non-specified transformation industries					
Iron and steel					
Non-ferrous metals					
Chemicals					
Pulp and paper					
Food and tobacco					
Cement					
Other non-metallic minerals					
Transport equipment					
Machinery					
Mining and quarrying					
Wood products					
Construction					

Textile and leather	Paraffin waxes, Petroleum coke, Other oil products, Oil shale and oil sands)				
Other non-specified industries					
International aviation			None	Lognormal CV=12.5%	None
Domestic aviation					
Rail					
International navigation	Natural gas (Natural gas)				
Domestic navigation					
Pipeline transport	Biomass (Renewable municipal waste, Primary solid biofuels, Biogases, Bio gasoline, Biodiesels, Bio jet kerosene, Other liquid biofuels, Non-specified primary biofuels/waste, Charcoal)				
Other non-specified transport					
Agriculture and forestry					
Fishing					
Cars			Uniform ±30%	Lognormal CV=12.5%	None
Light duty trucks					
Buses					
Heavy duty trucks					
Motorcycles					
Other fleet totals			None		
Commercial and institutional			Uniform ±30%	Lognormal CV=12.5% for regions constrained by locally measured OC emission factors or BBOA concentration measurements and CV=15% for other regions	None
Residential (rural)					
Residential (urban)					
Non-specified sectors			None		
Fugitive (solid fuel production)	Coke oven coke production	Normal 1970-1999 OECD countries CV=15%; non-OECD countries with local data fusion CV=20%; other countries CV=25% 2000-2020 OECD countries	None	Lognormal CV=12.5%	Uniform ±30%
Fugitive (oil and gas)	Oil production			Lognormal CV=12.5%	Uniform ±30%
	Oil storage and transport				
	Oil refining				
	Gasoline storage				
	Gasoline distribution				
	Diesel storage				
	Venting and flaring				
	Flaring in oil				

	refinery	CV=10%; non-OECD countries with local data fusion CV=15%; other countries CV=20%			
	Natural gas production, processing, storage, and distribution				
	Gas venting and flaring				
Mineral industry	Cement production	Normal 1970-1999 Developed countries CV=15% Other countries CV=20% 2000-2020 Developed countries CV=10% Other countries CV=15%	None	Lognormal CV=12.5%	Uniform ±30%
	Clicker production				
	Glass production				
	Lime production				
	Brick production				
Chemical industry (inorganic)	Ammonia		None	Lognormal CV=12.5%	Uniform ±30%
	Carbon black				
Chemical industry (organic)	Adipic acid		None		
	Ethylene				
	Vinyl chloride				
	Styrene				
	Low density polyethylene (LDPE)				
	High density polyethylene (HDPE)				
	Polyvinyl chloride (PVC)				
	Polypropylene (PP)				
	Polystyrene (PS)				
	SAN & ABS resins				
	Other synthetic resins				
	Ethylene oxide				
	Methanol				
	Acrylonitrile				
	Glyoxylic acid				
Chemical industry (manufacturing)	Synthetic rubber		None		
	Tyre production				
	Pharmaceutical production				
	Asphalt				
	Paint production				
	Printing ink production				

	Glues production				
	Shoes production				
	Leather tanning				
	Synthetic fibre				
	Wool				
	Silk				
	Cloth				
	Artificial fibre				
Metal industry	Sinter production		None	Lognormal CV=12.5%	Uniform ±30%
	Pellet production				
	Pig iron production				
	DRI production				
	Steel production		Uniform ±30%		
Pulp, paper, and food industry	Paper pulp		None	Lognormal CV=12.5%	Uniform ±30%
	Paper				
	Plywood				
	Bread				
	Biscuit				
	Sugar				
	Flour				
	Oilseed				
	Beer				
	Wine				
	Spirit				
Paint use	Architectural interior wall	Normal 1970-1999 Developed countries CV=25% Other countries CV=30% 2000-2020 Developed countries CV=15% Other countries CV=20%	Uniform ±30%	Uniform ±100%	Uniform ±30%
	Architecture other				
	Vehicle manufacturing				
	Vehicle repairing				
	Wood				
	Other industrial paint				
Other industrial use	Vehicle dewax		None		Uniform ±30%
	Vehicle reseal				
	Printing ink use				
	Glues and adhesives use				
	Preservation of wood				
	Degreasing				
	Dry cleaning				

Domestic use	Domestic use<br>Pesticide	None	Uniform<br>±30%

Table S6. Global and regional uncertainties of emission estimates.

		1970	1990	2010	2020	1970	1990	2010	2020
	Region	Lower bound				Upper bound			
L/SVOC	China	-76%	-75%	-54%	-46%	+88%	+81%	+57%	+51%
	India	-69%	-70%	-52%	-53%	+75%	+71%	+60%	+56%
	Rest of Asia	-69%	-65%	-53%	-50%	+76%	+71%	+57%	+55%
	Africa	-74%	-75%	-60%	-58%	+86%	+86%	+71%	+69%
	Canada and the U.S.	-53%	-47%	-40%	-36%	+63%	+57%	+50%	+47%
	Latin America	-61%	-56%	-44%	-44%	+64%	+62%	+48%	+48%
	Western Europe	-47%	-47%	-43%	-43%	+57%	+54%	+51%	+53%
	Eastern Europe	-49%	-49%	-54%	-53%	+54%	+58%	+66%	+68%
	Oceania and Pacific	-59%	-51%	-44%	-46%	+70%	+60%	+57%	+60%
IVOC	China	-78%	-76%	-48%	-45%	+102%	+90%	+63%	+62%
	India	-70%	-71%	-54%	-54%	+84%	+83%	+69%	+68%
	Rest of Asia	-64%	-60%	-52%	-51%	+79%	+75%	+67%	+70%
	Africa	-73%	-74%	-60%	-58%	+89%	+88%	+73%	+72%
	Canada and the U.S.	-38%	-45%	-41%	-41%	+60%	+68%	+77%	+70%
	Latin America	-63%	-58%	-46%	-47%	+78%	+74%	+61%	+62%
	Western Europe	-43%	-44%	-43%	-45%	+69%	+70%	+75%	+74%
	Eastern Europe	-51%	-43%	-46%	-46%	+69%	+63%	+75%	+75%
	Oceania and Pacific	-51%	-51%	-43%	-46%	+76%	+81%	+70%	+70%

Table S7. Regional policies/legislation related to $PM_{2.5}$ emission control of industrial boilers.

Region	Year	Policy/Legislation
U.S.	1987	Standards of Performance for New Stationary Sources; Industrial Commercial-Institutional Steam Generating Units; Final Rule 1987
	2006	Standards of Performance for Industrial Commercial-Institutional Steam Generating Units; and Standards of Performance for Small Industrial Commercial-Institutional Steam Generating Units Final Rule, Amendments
	2012	Standards of Performance for Fossil-Fuel-Fired Electric Utility, Industrial-Commercial Institutional, and Small Industrial Commercial-Institutional Steam Generating Units Final Rule
EU	1988	Council Directive of 24 November 1988 on the limitation of emissions of certain pollutants into the air from large combustion plants (88/609/EEC)
	2012	Convention on Long-range Transboundary Air Pollution 1999 Protocol to Abate Acidification, Eutrophication and Ground-level Ozone, amended on 4 May 2012<br>Directive 2010/75/EU of the European Parliament and of the Council of 24 November 2010 on industrial emissions (integrated pollution prevention and control)
Russia	2000	Federal Law No. 96-FZ on the protection of the atmospheric air
Japan	1971	Act on Improvement of Pollution Prevention Systems in Specified Factories
	1993	Regulatory Measures against Air Pollutants Emitted from Factories and Business Sites and the Outline of Regulation
	2007	工場及び事業場から排出される大気汚染物質に対する規制方式とその概要
China	1983	GB 3841-1983 Standard of smoke and dust emission for boiler
	2001	GB 13271-2001 Emission standard of air pollutants for coal-burning oil-burning gas-fired boiler
	2014	GB 13271-2014 Emission standard of air pollutants for boiler
	2018	Technical Guidelines for Feasible Pollution Control Technologies for Industrial Boilers (Draft for Comments)
India	1986	The Environment (Protection) Rules, 1986
	2014	Environment (Protection) (Fifth Amendment) Rules, 2014
Brazil	2006	CONAMA Resolution 382/06
Turkey	1986	Air Quality Assurance Regulation (AQAR)
	2004	Industrial Air Pollution Regulation
	2009	Regulation on air pollution caused by industry
South Africa	2004	National Environmental Management: Air Quality Act 39 of 2004
Egypt	2001	The National Environmental Action Plan of Egypt

Table S8. OC unabated emission factors.

Major sector	Source	Region	Reference	Value (g/kg)
Biofuel combustion	Residential	China	MEIC [49]	4.0 (crop)<br>4.5 (wood)
		South Asia	Parashar et al., 2005 [45]; Saud et al., 2012 [46]; Pandey et al., 2017 [47];	4.9
		Africa	DACCIWA [41]	8.5
Fugitive and industrial process	Coke	Global	GID-Iron and steel [1,48]	0.54
	Sinter/Pellet			0.026 (end of pipe)<br>0.005 (fugitive)
	Steel			2.1 (BOF)<br>0.18 (EAF)<br>1.3 (OHF)
	Cement		GID-Cement [10,44]	0.022
	Lime		MEIC [49]; Klimont et al., 2002 [42]; Kupiainen and Klimont, 2004 [43]	0.014
	Brick			0.004-0.09*

* Depend on brick kiln types.

Table S9. Observed-to-simulated BBOA ratios in Europe.

Sub-region	Country/Territory	Ratio
Western Europe	Austria, Belgium, France, Germany, Ireland, Isle of Man, Jersey, Liechtenstein, Luxembourg, Netherlands, Switzerland, United Kingdom	4.0
Eastern Europe	Albania, Belarus, Bosnia and Herzegovina, Bulgaria, Croatia, Czech Republic, Estonia, Hungary, Latvia, Lithuania, Moldova, Montenegro, North Macedonia, Poland, Romania, Serbia, Slovakia, Slovenia, Ukraine	6.0
Northern Europe	Denmark, Faeroe Islands, Finland, Greenland, Iceland, Norway, Sweden	1.0
Southern Europe	Cyprus, Gibraltar, Greece, Italy, Malta, Portugal, Spain	6.0

Table S10. Source mapping of emission control.

Source	Mapped to
Fugitive-Coke	Combustion-Industrial boiler
Industrial process-Sinter/Pellet	Combustion-Iron and steel
Industrial process-Steel	Combustion-Iron and steel
Industrial process-Cement	Combustion-Cement
Industrial process-Lime	Combustion-Industrial boiler
Industrial process-Brick	Combustion-Industrial boiler

Table S11. Proportions of emission factors by number with different confidence levels for each region and major sector. Values in parentheses represent their contributions to total L/S/IVOC emissions in the corresponding region and sector in 2020.

Region	Confidence level[a]	Coal combustion	Oil combustion	Biofuel combustion	Fugitive and industrial process	VCPs use	Other sources
China	*A+*	33.3% **(94.1%)**	58.8% **(70.8%)**	33.3% **(99.7%)**	0%	15.4% **(28.2%)**	0%
	A	0%	0%	0%	0%	0%	0%
	B	0%	5.9% **(1.0%)**	0%	0%	76.9% **(71.1%)**	0%
	C	0%	5.9% **(0.3%)**	0%	100% **(100%)**	0%	0%
	D	66.7% **(5.9%)**	29.4% **(27.9%)**	66.7% **(0.3%)**	0%	7.7% **(0.7%)**	100% **(100%)**
India	*A+*	0%	0%	33.3% **(79.7%)**	0%	0%	0%
	A	0%	0%	0%	0%	0%	0%
	B	33.3% **(86.5%)**	64.7% **(38.6%)**	0%	0%	92.3% **(99.8%)**	0%
	C	0%	5.9% (0.04%)	0%	100% **(100%)**	0%	0%
	D	66.7% **(13.5%)**	29.4% **(61.4%)**	66.7% **(20.3%)**	0%	7.7% **(0.2%)**	100% **(100%)**
Rest of Asia	*A+*	0%	0%	0%	0%	0%	0%
	A	0%	0%	0%	0%	0%	0%
	B	33.3% **(84.2%)**	64.7% **(41.8%)**	33.3% **(84.5%)**	0%	92.3% **(99.3%)**	0%
	C	0%	5.9% **(0.1%)**	0%	100% **(100%)**	0%	0%
	D	66.7% **(15.8%)**	29.4% **(58.1%)**	66.7% **(15.5%)**	0%	7.7% **(0.7%)**	100% **(100%)**
Africa	*A+*	0%	0%	0%	0%	0%	0%
	A	0%	0%	33.3% **(98.8%)**	0%	0%	0%
	B	33.3% **(84.8%)**	64.7% **(53.4%)**	0%	0%	92.3% **(99.4%)**	0%

	C	0%	5.9% **(0.1%)**	0%	100% **(100%)**	0%	0%
	D	66.7% **(15.2%)**	29.4% **(46.5%)**	66.7% **(1.2%)**	0%	7.7% **(0.6%)**	100% **(100%)**
**Canada and the U.S.**	***A+***	0%	58.8% **(53.2%)**	33.3% **(56.4%)**	0%	0%	0%
	A	0%	0%	0%	0%	0%	0%
	B	33.3% **(19.5%)**	5.9% **(26.6%)**	0%	0%	92.3% **(98.6%)**	0%
	C	0%	5.9% **(1.7%)**	0%	100% **(100%)**	0%	0%
	D	66.7% **(80.5%)**	29.4% **(18.5%)**	66.7% **(43.6%)**	0%	7.7% **(1.4%)**	100% **(100%)**
**Latin America**	***A+***	0%	0%	0%	0%	0%	0%
	A	0%	0%	0%	0%	0%	0%
	B	33.3% **(21.8%)**	64.7% **(67.3%)**	33.3% **(51.6%)**	0%	92.3% **(99.2%)**	0%
	C	0%	5.9% **(0.1%)**	0%	100% **(100%)**	0%	0%
	D	66.7% **(78.2%)**	29.4% **(32.5%)**	66.7% **(48.4%)**	0%	7.7% **(0.8%)**	100% **(100%)**
**Western Europe**	***A+***	0%	0%	0%	0%	0%	0%
	A	0%	0%	33.3% **(81.1%)**	0%	0%	0%
	B	33.3% **(64.6%)**	64.7% **(83.4%)**	0%	0%	92.3% **(97.4%)**	0%
	C	0%	5.9% **(0.3%)**	0%	100% **(100%)**	0%	0%
	D	66.7% **(35.4%)**	29.4% **(16.4%)**	66.7% **(16.9%)**	0%	7.7% **(2.6%)**	100% **(100%)**
**Eastern Europe**	***A+***	0%	0%	0%	0%	0%	0%
	A	0%	0%	33.3% **(93.6%)**	0%	0%	0%
	B	33.3% **(88.3%)**	64.7% **(87.0%)**	0%	0%	92.3% **(98.2%)**	0%
	C	0%	5.9% **(0.3%)**	0%	100% **(100%)**	0%	0%
	D	66.7% **(11.7%)**	29.4% **(12.7%)**	66.7% **(6.4%)**	0%	7.7% **(1.8%)**	100% **(100%)**
**Oceanic**	***A+***	0%	0%	0%	0%	0%	0%

**and Pacific**	***A***	0%	0%	0%	0%	0%	0%
	B	33.3% **(15.7%)**	64.7% **(81.2%)**	33.3% **(76.4%)**	0%	92.3% **(98.3%)**	0%
	C	0%	5.9% **(2.2%)**	0%	100% **(100%)**	0%	0%
	D	66.7% **(84.3%)**	29.4% **(16.7%)**	66.7% **(23.6%)**	0%	7.7% **(1.7%)**	100% **(100%)**

[a] A+: EFs measured locally; A: EFs measured in other regions but constrained by locally measured POA EFs or field observations; B: EFs measured in other regions without any constraints, or EFs derived by mass-balance-based estimates; C: EFs calculated from OC and NMVOC EFs; D: EFs lacking available data and taken from estimated values in literatures.

Table S12. Global and regional changes of L/SVOC, IVOC, and NMVOC emissions when including and excluding the year of 2020 in the trend analysis.

**Changes (%)**	**L/SVOC 1970-2020**	**L/SVOC 1970-2019**	**IVOC 1970-2020**	**IVOC 1970-2019**	**NMVOC 1970-2020**	**NMVOC 1970-2019**
Global	+59.0%	+62.0%	+72.2%	78.1%	+50.0%	+58.4%
China	-19.6%	-14.9%	+16.6%	+20.5%	+217.0%	+225.5%
India	+105.8%	+107.0%	+206.8%	+214.4%	+395.0%	+427.4%
Rest of Asia	+67.8%	+74.8%	+154.6%	+170.2%	+166.3%	+184.0%
Africa	+216.4%	+212.0%	+228.9%	+224.9%	+255.8%	+267.0%
Canada and the U.S.	-37.0%	-22.5%	-20.3%	-12.1%	-64.3%	-60.0%
Latin America	+72.5%	+75.6%	+83.5%	+95.2%	+163.0%	+181.7%
Western Europe	-20.1%	-11.8%	+13,0%	+20.4%	-68.8%	-66.3%
Eastern Europe	-4.7%	-4.4%	-3.1%	-1.2%	-16.5%	-12.3%
Oceanic and Pacific	-47.2%	-44.9%	-1.0%	+3.0%	-26.3%	-25.0%

Table S13. Global and regional growth rates of L/SVOC, IVOC, and NMVOC emissions when including and excluding the year of 2020 in the trend analysis.

**Growth rate (% per decade)**	**L/SVOC 1970-2020**	**L/SVOC 1970-2019**	**IVOC 1970-2020**	**IVOC 1970-2019**	**NMVOC 1970-2020**	**NMVOC 1970-2019**
Global	+9.7%	+10.3%	+11.5%	+12.5%	+8.4%	+9.8%
China	-4.3%	-3.2%	+3.1%	+3.9%	+26.0%	+27.2%
India	+15.5%	+16.0%	+25.1%	+26.3%	+37.7%	+40.4%
Rest of Asia	+10.9%	+12.1%	+20.6%	+22.5%	+21.6%	+23.7%
Africa	+25.9%	+26.1%	+26.9%	+27.2%	+28.9%	+30.4%
Canada and the U.S.	-8.8%	-5.1%	-4.4%	-2.6%	-18.6%	-17.1%
Latin America	+11.5%	+12.2%	+12.9%	+14.6%	+21.3%	+23.5%
Western Europe	-4.4%	-2.5%	+2.5%	+3.9%	-20.8%	-19.9%
Eastern Europe	-1.0%	-0.9%	-0.6%	-0.3%	-3.5%	-2.6%
Oceanic and Pacific	-12.0%	-11.4%	-0.2%	+0.6%	-5.9%	-5.7%

Table S14. Global emission changes and growth rates of L/SVOC, IVOC, and NMVOC emissions under volatility definitions shifted by one bin toward higher or lower volatility.

		Base	***Higher*** **One bin shifting towards higher volatility**	***Lower*** **One bin shifting towards lower volatility**
**Changes (%) 1970-2020**	L/SVOC	+59.0%	+57.9%	+62.3%
	IVOC	+72.2%	+68.2%	+61.5%
	NMVOC	+50.0%	+50.0%	+53.5%
**Growth rate (% per decade) 1970-2020**	L/SVOC	+9.7%	+9.6%	+10.2%
	IVOC	+11.5%	+11.0%	+10.1%
	NMVOC	+8.4%	+8.4%	+9.0%

Table S15. Global emission changes and growth rates of L/SVOC and IVOC emissions under *η_High* and *η_Low* cases.

		Base	***η_High***	***η_Low***
**Changes (%) 1970-2020**	L/SVOC	+59.0%	+58.8%	+85.2%
	IVOC	+72.2%	+67.8%	+81.5%
**Growth rate (% per decade) 1970-2020**	L/SVOC	+9.7%	+9.7%	+13.1%
	IVOC	+11.5%	+10.9%	+12.7%

Table S16. Comparison of model performance among the simulations using MEIC-global-FV, CEDS, and CEDS+IVOC as emission input.

		**NMB**	**NME**	**RMSE**	**r**
MEIC-global-FV	OC	-15.4%	34.0%	2.0	0.8
	POA	-29.4%	58.2%	3.9	0.8
	SOA	-21.4%	46.6%	4.5	0.6
CEDS	OC	-29.0%	40.3%	2.2	0.8
	POA	-1.0%	61.6%	3.5	0.8
	SOA	-70.8%	74.0%	6.5	0.5
CEDS+IVOC	OC	-26.7%	39.1%	2.2	0.8
	POA	-1.0%	61.6%	3.5	0.8
	SOA	-68.4%	72.0%	6.4	0.5

References

1. Xu R, Tong D, Davis SJ, et al. Plant-by-plant decarbonization strategies for the global steel industry. Nat Clim Change 2023;13(10):1067-1074.

2. Xu R, Ma H, Li J, et al. A technology-based global non-methane volatile organic compounds (NMVOC) emission inventory under the MEIC framework. Atmos Chem Phys 2025;25(19):12675-12700.

3. Hodzic A, Kasibhatla PS, Jo DS, et al. Rethinking the global secondary organic aerosol (SOA) budget: stronger production, faster removal, shorter lifetime. Atmos Chem Phys 2016;16(12):7917-7941.

4. Miao R, Xu R, Huang S, et al. Revisiting global organic aerosol budget based on a united modeling framework for semivolatile and intermediate-volatility organic compounds. Submitted 2025.

5. Jo DS, Park RJ, Kim MJ, et al. Effects of chemical aging on global secondary organic aerosol using the volatility basis set approach. Atmos Environ 2013;81:230-244.

6. Chang X, Zhao B, Zheng H, et al. Full-volatility emission framework corrects missing and underestimated secondary organic aerosol sources. One Earth 2022;5(4):403-412.

7. Huo Y, Guo Z, Liu Y, et al. Addressing unresolved complex mixture of I/SVOCs emitted from incomplete combustion of solid fuels by nontarget analysis. J Geophys Res Atmos 2021;126(23):e2021JD035835.

8. Cai S, Zhu L, Wang S, et al. Time-resolved intermediate-volatility and semivolatile organic compound emissions from household coal combustion in Northern China. Environ Sci Technol 2019;53(15):9269-9278.

9. Tong D, Geng G, Zhang Q, et al. Health co-benefits of climate change mitigation depend on strategic power plant retirements and pollution controls. Nat Clim Change 2021;11(12):1077-1083.

10. Yan X, Tong D, Cao Z, et al. Historical air pollutant emissions and future sustainable pathways of global cement plants. Resour Conserv Recycl 2024;211:107896.

11. Zhao Y, Nguyen NT, Presto AA, et al. Intermediate volatility organic compound emissions from on-road diesel vehicles: chemical composition, emission factors, and estimated secondary organic aerosol production. Environ Sci Technol 2015;49(19):11516-11526.

12. Zhao Y, Nguyen NT, Presto AA, et al. Intermediate volatility organic compound emissions from on-road gasoline vehicles and small off-road gasoline engines. Environ Sci Technol 2016;50(8):4554-4563.

13. Liu Y, Li Y, Yuan Z, et al. Identification of two main origins of intermediate-volatility organic compound emissions from vehicles in China through two-phase simultaneous characterization. Environ Pollut 2021;281:117020.

14. He X, Zheng X, You Y, et al. Comprehensive chemical characterization of gaseous I/SVOC emissions from heavy-duty diesel vehicles using two-dimensional gas chromatography time-of-flight mass spectrometry. Environ Pollut 2022;305:119284.

15. Zhang X, He X, Cao Y, et al. Comprehensive characterization of speciated volatile organic compounds (VOCs), gas-phase and particle-phase intermediate-and semi-volatile volatility organic compounds (I/S-VOCs) from Chinese diesel trucks. Sci Total Environ 2024;912:168950.

16. Wu Y, Zhang S, Hao J, et al. On-road vehicle emissions and their control in China: A review and outlook. Sci Total Environ 2017;574:332-349.

17. Qi L, Liu H, Shen XE, et al. Intermediate-volatility organic compound emissions from nonroad construction machinery under different operation modes. Environ Sci Technol 2019;53(23):13832-13840.

18. Shen X, Che H, Yao Z, et al. Real-world emission characteristics of full-volatility organics originating from nonroad agricultural machinery during agricultural activities. Environ Sci Technol 2023;57(28):10308-10318.

19. Huang C, Hu Q, Li Y, et al. Intermediate volatility organic compound emissions from a large cargo vessel operated under real-world conditions. Environ Sci Technol 2018;52(21):12934-12942.

20. Jathar SH, Miracolo MA, Presto AA, et al. Modeling the formation and properties of traditional and non-traditional secondary organic aerosol: problem formulation and application to aircraft exhaust. Atmos Chem Phys 2012;12(19):9025-9040.

21. Sinha A, George I, Holder A, et al. Development of volatility distributions for organic matter in biomass burning emissions. Environ Sci Atmos 2023;3(1):11-23.

22. Huo Y, Guo Z, Liu Y, et al. Addressing unresolved complex mixture of I/SVOCs emitted from incomplete combustion of solid fuels by nontarget analysis. J Geophys Res Atmos 2021;126(23):e2021JD035835.

23. Huang G, Wang S, Chang X, et al. Emission factors and chemical profile of I/SVOCs emitted from household biomass stove in China. Sci Total Environ 2022;842:156940.

24. Stewart GJ, Nelson BS, Acton WJF, et al. Comprehensive organic emission profiles, secondary organic aerosol production potential, and OH reactivity of domestic fuel combustion in Delhi, India. Environ Sci Atmos 2021;1(2):104-117.

25. Stewart GJ, Nelson BS, Acton WJF, et al. Emission estimates and inventories of non-methane volatile organic compounds from anthropogenic burning sources in India. Atmos Environ X 2021;11:100115.

26. Kurokawa J, Ohara T. Long-term historical trends in air pollutant emissions in Asia: Regional Emission inventory in ASia (REAS) version 3. Atmos Chem Phys 2020;20(21):12761-12793.

27. Hoesly RM, Smith SJ, Feng L, et al. Historical (1750–2014) anthropogenic emissions of reactive gases and aerosols from the Community Emissions Data System (CEDS). Geosci Model Dev 2018;11(1):369-408.

28. Denier van der Gon H, Bergström R, Fountoukis C, et al. Particulate emissions from residential wood combustion in Europe–revised estimates and an evaluation. Atmos Chem Phys 2015;15(11):6503-6519.

29. Donahue NM, Robinson AL, Stanier CO, et al. Coupled partitioning, dilution, and chemical aging of semivolatile organics. Environ Sci Technol 2006;40(8):2635-2643.

30. Jiang J, Aksoyoglu S, El-Haddad I, et al. Sources of organic aerosols in Europe: a modeling study using CAMx with modified volatility basis set scheme. Atmos Chem Phys 2019;19(24):15247-15270.

31. Ciarelli G, Aksoyoglu S, El Haddad I, et al. Modelling winter organic aerosol at the European scale with CAMx: evaluation and source apportionment with a VBS parameterization based on novel wood burning smog chamber experiments. Atmos Chem Phys 2017;17(12):7653-7669.

32. Daellenbach KR, Manousakas M, Jiang J, et al. Organic aerosol sources in the Milan metropolitan area–Receptor modelling based on field observations and air quality modelling. Atmos Environ 2023;307:119799.

33. Huang L, Zhao B, Wang S, et al. Global anthropogenic emissions of full-volatility organic compounds. Environ Sci Technol 2023;57(43):16435-16445.

34. Yang X, Song K, Guo S, et al. Elucidating the unexpected importance of intermediate-volatility organic compounds (IVOCs) from refueling procedure. J Hazard Mater 2024;471:134361.

35. Simon H, Beck L, Bhave PV, et al. The development and uses of EPA's SPECIATE database. Atmos Pollut Res 2010;1(4):196-206.

36. Mansouri K, Grulke CM, Judson RS, et al. OPERA models for predicting physicochemical properties and environmental fate endpoints. J Cheminf 2018;10:1-19.

37. Liang C, Feng B, Wang S, et al. Differentiated emissions and secondary organic aerosol formation potential of organic vapor from industrial coatings in China. J Hazard Mater 2024;466:133668.

38. McDonald BC, De Gouw JA, Gilman JB, et al. Volatile chemical products emerging as largest petrochemical source of urban organic emissions. Science 2018;359(6377):760-764.

39. Tanzer-Gruener R, Rajan PE, Dugan LD, et al. Watching paint dry: organic vapor emissions from architectural coatings and their impact on secondary organic aerosol formation. Environ Sci Technol 2022;56(16):11236-11245.

40. Seltzer KM, Pennington E, Rao V, et al. Reactive organic carbon emissions from volatile chemical products. Atmos Chem Phys 2021;21(6):5079-5100.

41. Keita S, Liousse C, Assamoi EM, et al. African anthropogenic emissions inventory for gases and particles from 1990 to 2015. Earth Syst Sci Data 2021;13(7):3691-3705.

42. Klimont Z, Cofala J, Bertok I, et al. Modeling particulate emissions in Europe. A framework to estimate reduction potential and control costs. https://pure.iiasa.ac.at/id/eprint/6712/ 2002.

43. Kupiainen K, Klimont Z. Primary emissions of submicron and carbonaceous particles in Europe and the potential for their control. https://pure.iiasa.ac.at/id/eprint/7371/ 2004.

44. Liu J, Tong D, Zheng Y, et al. Carbon and air pollutant emissions from China's cement industry 1990–2015: trends, evolution of technologies, and drivers. Atmos Chem Phys 2021;21(3):1627-1647.

45. Parashar DC, Gadi R, Mandal TK, et al. Carbonaceous aerosol emissions from India. Atmos Environ 2005;39(40):7861-7871.

46. Saud T, Gautam R, Mandal TK, et al. Emission estimates of organic and elemental carbon from household biomass fuel used over the Indo-Gangetic Plain (IGP), India. Atmos Environ 2012;61:212-220.

47. Pandey A, Patel S, Pervez S, et al. Aerosol emissions factors from traditional biomass cookstoves in India: insights from field measurements. Atmos Chem Phys 2017;17(22):13721-13729.

48. Wang X, Lei Y, Yan L, et al. A unit-based emission inventory of SO_2, NO_x and PM for the Chinese iron and steel industry from 2010 to 2015. Sci Total Environ 2019;676:18-30.

49. Zheng B, Tong D, Li M, et al. Trends in China's anthropogenic emissions since 2010 as the consequence of clean air actions. Atmos Chem Phys 2018;18(19):14095-14111.

50. Bilde M, Barsanti K, Booth M, et al. Saturation vapor pressures and transition enthalpies of low-volatility organic molecules of atmospheric relevance: from dicarboxylic acids to complex mixtures. Chem Rev 2015;115(10):4115-4156.

51. Bey I, Jacob DJ, Yantosca RM, et al. Global modeling of tropospheric chemistry with assimilated meteorology: Model description and evaluation. J Geophys Res Atmos 2001;106:23073-23095.

52. Wang Y, Jacob DJ, Logan JA, et al. Global simulation of tropospheric O_3-NO_x-hydrocarbon chemistry: 1. Model formulation. J Geophys Res Atmos 1998;103:10713-10725.

53. Zhang LM, Gong SL, Padro J, et al. A size-segregated particle dry deposition scheme for an atmospheric aerosol module. Atmos Environ 2001;35:549-560.

54. Liu H, Jacob DJ, Bey I, et al. Constraints from ^{210}Pb and ^{7}Be on wet deposition and transport in a global three-dimensional chemical tracer model driven by assimilated meteorological fields. J Geophys Res Atmos 2001;106:12109-12128.

55. Geng G, Zheng Y, Zhang Q, et al. Drivers of $PM_{2.5}$ air pollution deaths in China 2002–2017. Nat Geosci 2021;14(9):645-650.

56. Wiedinmyer C, Kimura Y, McDonald-Buller EC, et al. The Fire Inventory from NCAR version 2.5: an updated global fire emissions model for climate and chemistry applications. Geosci Model Dev 2023;16:3873-3891.

57. Guenther AB, Jiang X, Heald CL, et al. The Model of Emissions of Gases and Aerosols from Nature version 2.1 (MEGAN2.1): an extended and updated framework for modeling biogenic emissions. Geosci Model Dev 2012;5:1471-1492.

58. Carter WPL. Development of an improved chemical speciation database for processing emissions of volatile organic compounds for air quality models. 2023. https://intra.engr.ucr.edu/~carter/emitdb/.

59. Li M, Zhang Q, Streets DG, et al. Mapping Asian anthropogenic emissions of non-methane volatile organic compounds to multiple chemical mechanisms. Atmos Chem Phys 2014;14(11):5617-5638.

60. WorldPop. https://www.worldpop.org/

61. History database of the Global Environment (HYDE). https://landuse.sites.uu.nl/datasets/

62. Liu H, Gong P, Wang J, et al. Annual dynamics of global land cover and its long-term changes from 1982 to 2015. Earth Syst Sci Data 2020;12(2):1217-1243.

63. OAG. https://www.oag.com/

64. OpenStreetMap. https://www.openstreetmap.org/

65. Liu H, Fu M, Jin X, et al. Health and climate impacts of ocean-going vessels in East Asia. Nat Clim Chang 2016;6(11):1037-1041.

66. Wang XT, Liu H, Lv ZF, et al. Trade-linked shipping CO_2 emissions. Nat Clim Chang 2021;11(11):945-951.

67. Huang RJ, Li YJ, Chen Q, et al. Secondary organic aerosol in urban China: A distinct chemical regime for air pollution studies. Science 2025;389(6763):eadq2840.

68. Pye HOT, Seinfeld JH. A global perspective on aerosol from low-volatility organic compounds. Atmos Chem Phys 2010;10(9):4377-4401.

69. Pye HOT, Chan AWH, Barkley MP, et al. Global modeling of organic aerosol: the importance of reactive nitrogen (NO_x and NO_3). Atmos Chem Phys 2010;10(22):11261-11276.
